# Joint analysis of quantitative trait loci and major-effect causative mutations affecting meat quality and carcass composition traits in pigs

**DOI:** 10.1186/1471-2156-12-76

**Published:** 2011-08-29

**Authors:** Pierre Cherel, José Pires, Jérôme Glénisson, Denis Milan, Nathalie Iannuccelli, Frédéric Hérault, Marie Damon, Pascale Le Roy

**Affiliations:** 1 Hendrix-Genetics RTC, 100 avenue Denis-Papin, St-Jean-de-Braye, France; 2 INRA, UR444 Laboratoire de Génétique Cellulaire, 31320 Castanet-Tolosan, France; 3 INRA, UMR0598, Génétique Animale, 35042 Rennes cedex, France; 4 Agrocampus Ouest, UMR0598, Génétique Animale, 35042 Rennes cedex, France; 5 INRA, UMR1079 SENAH, 35590 Saint-Gilles, France; 6 Agrocampus Ouest, UMR1079 SENAH, 35590 Saint-Gilles, France

## Abstract

**Background:**

Detection of quantitative trait loci (QTLs) affecting meat quality traits in pigs is crucial for the design of efficient marker-assisted selection programs and to initiate efforts toward the identification of underlying polymorphisms. The RYR1 and PRKAG3 causative mutations, originally identified from major effects on meat characteristics, can be used both as controls for an overall QTL detection strategy for diversely affected traits and as a scale for detected QTL effects. We report on a microsatellite-based QTL detection scan including all autosomes for pig meat quality and carcass composition traits in an F2 population of 1,000 females and barrows resulting from an intercross between a Pietrain and a Large White-Hampshire-Duroc synthetic sire line. Our QTL detection design allowed side-by-side comparison of the RYR1 and PRKAG3 mutation effects seen as QTLs when segregating at low frequencies (0.03-0.08), with independent QTL effects detected from most of the same population, excluding any carrier of these mutations.

**Results:**

Large QTL effects were detected in the absence of the RYR1 and PRKGA3 mutations, accounting for 12.7% of phenotypic variation in loin colour redness CIE-a* on SSC6 and 15% of phenotypic variation in glycolytic potential on SSC1. We detected 8 significant QTLs with effects on meat quality traits and 20 significant QTLs for carcass composition and growth traits under these conditions. In control analyses including mutation carriers, RYR1 and PRKAG3 mutations were detected as QTLs, from highly significant to suggestive, and explained 53% to 5% of the phenotypic variance according to the trait.

**Conclusions:**

Our results suggest that part of muscle development and backfat thickness effects commonly attributed to the RYR1 mutation may be a consequence of linkage with independent QTLs affecting those traits. The proportion of variation explained by the most significant QTLs detected in this work is close to the influence of major-effect mutations on the least affected traits, but is one order of magnitude lower than effect on variance of traits primarily affected by these causative mutations. This suggests that uncovering physiological traits directly affected by genetic polymorphisms would be an appropriate approach for further characterization of QTLs.

## Background

Early research into the genetics of meat quality in pigs found causative mutations associated with marked effects on pig meat value, leading to worldwide applications in the pork production chain. The RYR1 Cys^615 ^mutation [1] alters the function of a skeletal muscle sarcoplasmic calcium release channel, changes the contraction response to stimuli and gives rise to a malignant hyperthermia syndrome (MHS) on exposure to halothane gas. Characterization of this recessive phenotype first identified homozygous mutants, and subsequently the mutation itself. This mutation increases the pH fall rate in skeletal muscle *post mortem *and leads to the production of pale, soft, exsudative (PSE) meat, a long-recognized meat defect associated with malignant hyperthermia [2].

The PRKAG3 Gln^200 ^[3] mutation causes a dominant gain of function in adenosine monophosphate activated protein kinase (AMPK) complex regulating energy balance in skeletal muscle cells, which leads to an increased glycogen content of skeletal muscle tissue associated with an extended muscle pH fall *post mortem *and meat acidification. Measurements of muscle and meat glycogen concentrations, along with glycogen metabolites, were instrumental in the identification of mutation carriers and assisted the positional cloning of the causative mutation [3].

Outside these two causative mutations, phenotypic and genetic variation in meat quality traits is typically broad and seldom selected for, as pedigree record acquisition through the logistics of animal transport, slaughtering and meat appraisal is extremely costly. Selection efficiency is also limited by inaccurate estimation of breeding values for typical non-slaughtered selection candidates, as it relies solely on slaughtered relatives' phenotypes. With the benefits of genetic marker-assisted selection in mind, several studies of quantitative trait loci (QTLs) have dissected the genetic variation of meat quality traits in a range of swine populations, initially in exotic crosses to maximize segregating variation (Meishan, Erhualian and wild boar [4-8]) and then in more typical pig breeds used in pork production (Large-White, Duroc and Pietrain [9-16]).

We set out to revisit QTL detection of meat quality traits in the context of the genetic variation in commercial pig lines, purposely including in our reference population a limited proportion of RYR1 and PRKAG3 mutation carriers to directly compare examples of causative polymorphisms identified by QTL positional cloning, and newly detected QTLs.

## Results and Discussion

### Detection of eight significant QTLs contributing to genetic variation in meat quality traits

We detected 8 QTLs significant at a 5% genome-wise level for 15 meat quality traits analyzed (listed in Table 1). These significant QTLs explain 9-23% of the phenotypic variance. Significant QTLs were detected for loin colour traits (4 QTLs), loin chemical composition (2 QTLs) and raw meat texture (2 QTLs). For completeness, we also report 30 suggestive QTLs for meat quality traits, found significant at a 1% or 5% chromosome-wise threshold. A genome position-sorted list of significant QTLs is presented in Table 2. A genome position-sorted list of suggestive QTLs is presented in Additional File 1.

**Table 1:** Meat quality, carcass and growth traits definition and summary

Abbreviation	Unit	*N*	mean	SD	Trait definition
LL-pH	pH unit	1023	5.71	0.20	LL pH, 24-h *post mortem*.
SM-pH	pH unit	1024	5.79	0.19	SM pH, 24-h *post mortem*.
Glyc-P	μmol/g	805	159.4	24.8	LL Glycolytic potential, 24 h
pH-45	pH unit	840	6.55	0.18	Loin pH, 45 min *post mortem*.
LL-L*	CIE L*	852	49.63	3.06	Lightness LL, 30-h *post mortem*
LL-a*	CIE a*	852	7.86	1.22	Red versus Green LL color
LL-b*	CIE b*	851	5.13	1.29	Yellow versus blue LL color
SF-cook	N	753	34.08	4.93	Shear force on cooked loin
SF-raw	N	792	36.04	6.35	Shear force on raw loin
IMF	%	804	2.24	0.66	Intramuscular fat %, LL
Drip-L	%	766	1.70	0.98	Loin drip losses, 48-h +4°C
Cook-Y	%	780	73.85	2.82	Loin cooking yield, %
SM-L*	CIE L*	769	51.41	3.99	Lightness SM, 96-h *post mortem*
SM-a*	CIE a*	769	10.19	1.84	Red versus green SM color
SM-b*	CIE b*	768	4.71	1.67	Yellow versus blue SM color
Birth-W	kg	1180	1.67	0.32	Individual birth weight
ADG	g/day	1022	1055	131	Average daily gain 35-105 kg
F-Ham	mm	869	12.99	2.97	Fat depth on ham cut section
F-US	mm	1022	13.36	2.59	Backfat depth, ultrasonic record
LMA-US	cm^2^	1023	45.20	5.49	Loin area, ultrasonic record
LMA-C	cm^2^	839	55.93	5.82	Loin eye area, on sliced chop
Ham-W	kg	1014	12.54	0.53	Ham weight, including feet
Loin-W	kg	1001	10.39	0.59	Loin weight, including bones
F-FOM-B	mm	1008	13.93	2.75	Fat depth, back last rib, F.O.M.
M-FOM-B	mm	1008	58.91	4.77	Loin depth, back last rib, F.O.M.
F-FOM-L	mm	1008	16.06	3.10	Fat depth, lumbar; F.O.M.
M-FOM-L	mm	1008	70.91	5.07	Loin depth, lumbar, F.O.M.
F-oP-B	mm	812	15.89	3.47	Fat depth, back last rib, Probe
F- oP -L	mm	812	19.43	3.92	Fat depth, lumbar, Probe
F-HCr-B	mm	868	19.16	3.09	Fat depth, back, ruler on cut
F- HCr -L	mm	868	26.25	3.91	Fat depth, lumbar, ruler on cut

Summary statistics refer to the population used for QTL detection, excluding RYR1 and PRKAG3 mutation carriers. LL: longissimus lumborum muscle; SM: semimembranosus muscle; FOM Fat-o-Meter carcass grading system.

**Table 2:** Significant QTLs detected for meat quality, carcass composition and growth traits

SSC	Trait	**Max (95% CI)**^**1**^	**LRT**^**2**^	**QTL v.**^**3**^	Flanking	markers
**1**	F-FOM-L	89 (65-97)	15.1 **	10.6%	S0122	Sw2185
	SF-raw	92 (72-112)	15.6 **	13.0%	S0122	Sw2185
	Glyc-P	111 (102-120)	12.5 *	15.9%	MC4R	FH2510
	F-Ham	137 (129-153)	34.4 **	12.7%	S0155	S0302

**3**	F-HCr-B	145 (137-153)	12.4 *	8.6%	S0002	FH1085

**5**	LL-L*	100 (72-188)	12.6 *	23.1%	Sj024	Sw453
	IMF	164 (144-180)	12.8 *	9.0%	S0005	Sw1468
	F-Ham	209 (193-245)	12.0 *	12.8%	IGF1	Swr378
	F-US	212 (164-232)	16.6 **	9.3%	IGF1	Swr378
	F-FOM-B	212 (196-240)	18.2 **	17.6%	IGF1	Swr378
	F-FOM-L	221 (201-237)	17.0 **	9.9%	IGF1	Swr378

**6**	LL-a*	54 (35-77)	20.2 **	12.4%	Sw1038	Sw1067
	LL-b*	64 (44-72)	16.3 **	9.5%	Sw1038	Sw1067
	F-oP-B	72 (52-88)	11.3 *	5.7%	Sw1067	Sw2521
	LMA-US	100 (80-108)	11.3 *	5.7%	Sw71	S0228

**7**	F-oP-B	88 (40-128)	12.0 *	8.9%	Sw1856	Sw1614

**8**	F-US	4 (0-16)	13.2 *	6.3%	Ks148	S0098
	Ham-W	52 (32-72)	13.8 *	6.4%	S0098	KS195

**10**	Birth-W	32 (4-48)	12.6 *	7.2%	Sw767	S0351

**11**	Ham-W	16 (0-36)	10.2 *	6.0%	Sw1460	Sw2008
	M-FOM-B	21 (0-41)	22.0 **	6.0%	Sw2008	Sw151
	LMA-US	36 (0-60)	10.0 *	5.8%	Sw2008	Sw151
	M-FOM-L	37 (13-61)	15.7 **	9.4%	Sw2008	Sw151

**13**	Loin-W	80 (72-92)	19.1 **	7.7%	Sw882	Sw129

**15**	F-US	32 (12-48)	26.8 **	8.1%	S0148	FH1710

**16**	SF-raw	16 (0-52)	11.0 *	9.2%	Sw1035	Sw1809
	F-FOM-B	64 (44-84)	12.7 *	9.1%	Swr2480	S0105

**18**	LL-L*	80 (68-80)	13.2 *	8.0%	Sw1682	FH1006

QTLs detected in F2 population excluding carriers of either RYR1 or PRKAG3 mutations. ^**1**^Position of most significant QTL detection on genetic map in cM (1-LOD drop-off confidence interval). **^2^**LRT significance levels: * 5% genome-wise; ** 1% genome-wise. ^**3**^Percentage of phenotypic variance associated with QTL effect.

### QTL confirmed for meat colour on SSC6

We detected a significant QTL for loin meat colour parameters (redness, yellowness) on SSC6 in a confidence interval overlapping confidence intervals reported for a QTL detected on this chromosome for loin redness a* in several populations: half-sib families generated in a four-way cross using Pietrain × Large White composite/Large White F1 sires [17] a Duroc × Pietrain F2 population [10], and a Duroc × Large White F2 population [16]. The QTL affecting a* in loin was the most significant of the meat quality QTLs detected in our study and explained 12% of the phenotypic variance. A QTL affecting the same trait, loin a* on SSC6 was also the most significant QTL reported from a QTL detection scan using a Japanese wild boar × Large White intercross population [8]. In this case, the NUDT7 gene was subsequently proposed as a candidate gene underlying this QTL, and alleles of this gene were described as associated with different NUDT7 transcription levels [18]. Recently, *in vitro *over-expression of this gene was shown to down-regulate heme synthesis, further substantiating claims for a biological mechanism leading to a meat colour change [19].

### QTL confirmed for glycolytic potential and ultimate pH on SSC1

**Figure 1:**
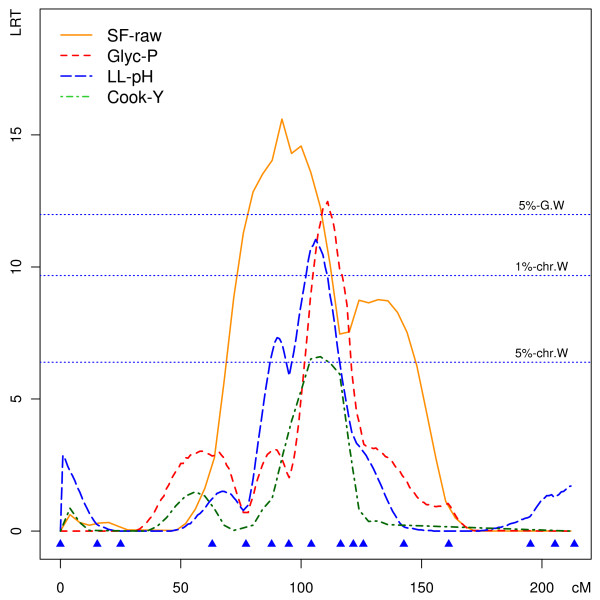
**LRT profiles for QTL detection on SSC1**. LRT profiles for QTL detection on SSC1, analyzing four loin meat quality traits: loin cooking yield (Cook-Y), glycolytic potential (Glyc-P), loin pH 24-h *post mortem *(LL-pH) and raw meat shear force (SF-raw). The genetic markers used are represented by filled blue triangles above *x*-axis (cM).

A major-effect QTL (15.9% phenotypic variance) was detected for glycolytic potential in loin on SSC1 with a maximum LRT between markers MC4R and FH2510. A QTL affecting glycolytic potential was reported within an overlapping confidence interval in an Erhualian × Duroc intercross [20]. At the same genomic location (or close to it), we detected a suggestive QTL for loin pH recorded 24 h *post mortem *(7.9% phenotypic variance), a suggestive QTL for loin cooking yield and a significant QTL (15.6% variance explained) for shear force of raw meat. QTL detection LRT profiles are plotted along the SSC1 genetic map for these four traits in Figure 1 and suggest a common determinism. QTL affecting ultimate pH were also reported in comparable genome locations in a Berkshire × Yorkshire F2 population [14] and in a Meishan × Large White backcross population [21], although confidence intervals defined in our study seem to fall outside of chromosome segments defined by recombination mapping in backcross experiments [21].

### QTL detected for intramuscular fat percentage

We detected a significant QTL affecting intramuscular fat on the distal q arm of SSC5. A QTL has been reported for both intramuscular fat loin content and loin marbling on the same chromosome segment in an Erhualian × Duroc population [7].

A suggestive QTL (1% chromosome-wise significance) for intramuscular fat percentage was detected on SSC6 in a confidence interval overlapping those estimated for QTLs affecting the same trait in different F2 populations: Iberic × Landrace [22], Duroc × Landrace [23], Duroc × Pietrain [10] and Pietrain × Synthetic [24]. If the QTLs detected in these different backgrounds are the same, then identified QTL alleles may be segregating in a large range of pig populations contributing to actual pork production and may reflect the lack of selection for this trait in many pig breeding programs, or antagonistic effects on other traits.

A third QTL affecting the same trait was detected on SSC7. Neighbouring locations on chromosome 7 were also suggested to contribute to genetic variation of this trait in a Duroc × Meishan F2 population [25] and in a Large White × Meishan F2 population [26]. This QTL was subsequently not confirmed as segregating within a purebred Duroc population [27]. The most likely location of this QTL in our study is between markers Sw2155 and LRA1, very close to the most likely location reported in the Large White × Meishan F2 population [26], but somewhere distant from the distal location proposed in the study of a Duroc × Meishan population [25]. Our population showed no contribution from Meishan alleles but may share Large White alleles with the Large White × Meishan study [26] or Duroc alleles with the Duroc × Meishan F2 population [25].

### QTL segregation for *post mortem *pH fall rate

We detected a suggestive QTL (1% chromosome-wise significance) on SSC12 affecting loin pH at 45 minutes *post mortem *between markers FH1993 and Sw957, where a QTL was detected for pH fall between 45 minutes and 3 hours *post mortem *in a Hampshire × Landrace cross [28]. This result may point to segregation of Hampshire breed derived alleles in both of these populations.

### Detection of 20 significant QTLs affecting carcass composition and growth traits

We detected 20 QTLs significant at a 5% genome-wise level for 16 carcass composition and growth traits analyzed and listed in Table 1. These significant QTLs explained 5.7-17.6% of phenotypic variance in our dataset. Significant QTLs were detected for fat thickness traits (11 QTLs), muscle development traits (7 QTLs) and birth weight (1 QTL). For completeness, we also report 49 suggestive QTLs for carcass and growth traits, significant at a 1% or 5% chromosome-wise threshold. A genome position-sorted list of significant QTL is presented in Table 2. A genome position-sorted list of suggestive QTL is presented in Additional File 1. These results point to several colocalized QTLs affecting fat deposition (5 QTLs on SSC5) or muscle development (4 QTLs on SSC11 and 4 QTLs on SSC13), suggesting pleiotropic effects of common determinants. Overlaid LRT profiles for detection of QTLs affecting these groups of traits on chromosomes 5, 11, and 13 are reported in Additional File 2.

A significant QTL was detected for birth weight on SSC10, where a QTL affecting the same trait was also reported at a similar position in a Duroc × Large White population [16]. This independent confirmation is noteworthy considering the low number of QTLs reported for this trait, and this result points to major-effect alleles (10% phenotypic variance in our population) segregating from Duroc and/or Large White breeds.

### PRKAG3 Gln^200 ^mutation detected as a QTL on SSC15

The causative major-effect PRKAG3 Gln^200 ^allele was detected as an extremely significant QTL explaining 53% of phenotypic variation in loin glycolytic potential in a control population including 3% of the mutated allele. Long-range linkage in this F2 population generates a significant QTL statistical test on most of SSC15. Highly significant QTLs were also detected at the same position for ultimate pH values in semimbranosus and longissimus muscles but not for pH-45, cooked loin shear force or intramuscular fat (Additional File 3). While QTL detection statistic at the PRKGA3 position on cooked loin shear force might be too low to reach significance thresholds in this population, the lack of any QTL detection affecting IMF or pH-45 suggests that previously reported significant genotype effects on these traits in association studies performed using this same dataset [29] could result from an association between genotype classes and undocumented class effects. Alternatively, the analysis of within-family segregation based on described IBD may have less power than population-wide comparison of genotype effects.

**Figure 2:**
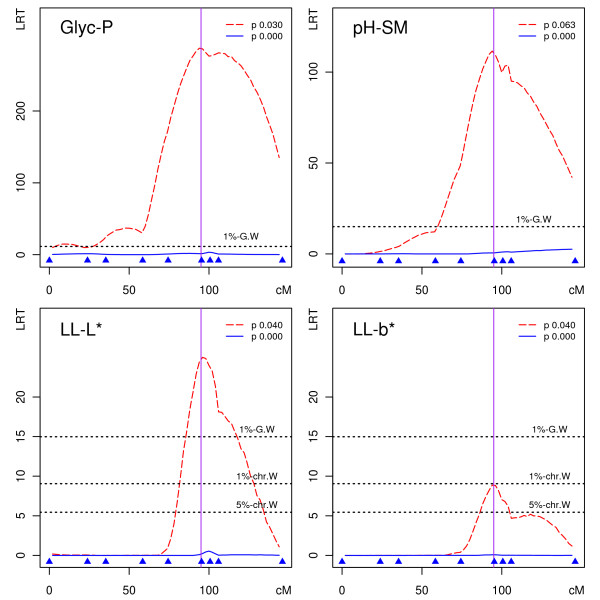
**Detection of the PRKAG3 Gln**^**200 **^**mutation as a QTL on SSC15**. LRT profiles for QTL detection on SSC15 within a population including (red dashed line) or excluding (blue continuous line) the PRKAG3 Gln^200 ^allele for four different traits (p as Gln^200 ^allele frequency). A vertical line is drawn at the PRKGA3 position on the SSC15 genetic map. The genetic markers used are represented by filled blue triangles above the *x*-axis (cM).

A QTL was also detected at the PRKAG3 position, but with a less extensive significance on the loin colour parameters lightness LL-L*, redness LL-a* and yellowness LL-b*, explaining 19%, 17% and 9% of phenotypic variance, respectively. Exclusion of mutation carriers from this control population completely abolished QTL detection for all of these traits, as summarized in Additional File 3. LRT detection profiles on SSC15 with or without mutation carriers are presented in Figure 2. While physiological effects of the PRKAG3 Gln^200 ^allele on muscle glycogen content have been experimentally validated in a murine knock-in model [30], our data also highlight a causative effect of PRKAG3 mutation on meat colour that cannot be analyzed in a mouse model and has been associated with PRKAG3 mutation carriers [31].

### RYR1 Cys^615 ^mutation detected as a QTL on SSC6

**Figure 3:**
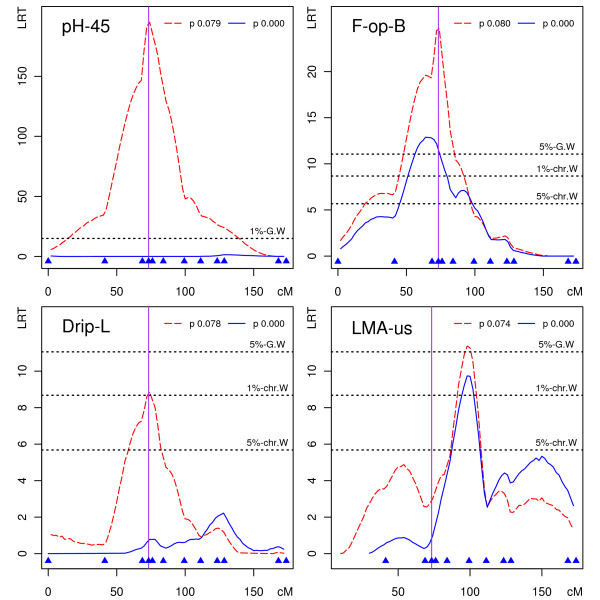
**Detection of the RYR1 Cys615 mutation as a QTL on SSC6**. LRT profiles for QTL detection on SSC6 within a population including (red dashed line) or excluding (blue continuous line) the RYR1 Cys^615 ^allele for four different traits (p as Cys^615 ^allele frequency). A vertical line is drawn at the RYR1 position on SSC6 genetic map. The genetic markers used are represented by filled blue triangles above the *x*-axis (cM).

The RYR1 Cys^615 ^mutation was detected as a highly significant QTL affecting pH-45 on SSC6, and explaining 35% of phenotypic variance in this population (mutant allele frequency, p Cys^615 ^= 0.08). Exclusion of mutation carriers resulted in a flat LRT profile for QTL detection for this trait on SSC6 as illustrated in Figure 3 and summarized in Additional File 3. A suggestive QTL was also detected at the RYR1 position on SSC6 affecting meat drip losses (Drip-L), and explained 5% of phenotypic variance in this dataset. As for pH-45, detection of this QTL did not persist on exclusion of mutation carriers (Figure 3 andAdditional File 3).

QTLs were also detected in this control population at the same or neighbouring locations (Figure 3) for a backfat thickness trait (F-op-B) and a loin muscle development trait (LMA-us). These results would fit generally accepted effects for the RYR1 mutation on carcass composition traits [32]. These apparent genotype effects have also been verified in this same dataset using a model of analysis fitting RYR1 genotypes as fixed effects in addition to a polygenic additive effect [29]. However, the exclusion of RYR1 mutation carriers from the QTL analysis does not stop detection of significant QTLs for those two carcass composition traits at the same chromosome locations (Figure 3, Additional File 3). For each of those two traits, our result suggests that in addition to the RYR1 mutation, an independent QTL located at a neighbouring location is segregating in this population. We also observed persisting suggestive or significant QTLs in the absence of the RYR1 mutation at similar positions on SSC6 when analyzing a related fatness trait (F-us) and a related loin muscle development trait (Loin-W), as summarized in Additional File 3.

**Figure 4:**
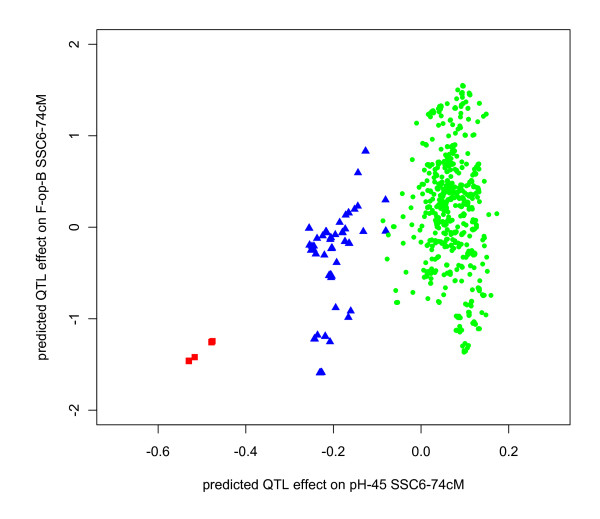
**Predicted individual QTL effect levels for pH-45 QTL (SSC6-74 cM) and F-op-B QTL (SSC6-74 cM) in the control F2 population including carriers of the RYR1 Cys**^**615 **^**mutation**. Individuals are plotted according to their RYR1 Cys^615 ^genotype (red squares are homozygous mutant animals, blue triangles are heterozygous carriers and green circles are wild type animals). Axes are drawn in character units: pH units on the *x*-axis, mm (fat depth) on the *y*-axis.

We indirectly inferred the most prevalent linkage phase associations in this population between the RYR1 alleles and the linked backfat or loin area QTL alleles through analyses of predicted QTL genotypic effects within RYR1 genotypes. Considering the expected extensive linkage between these two loci in this F2 population, association between RYR1 genotypes and QTL genotypes (and therefore QTL predicted effect) is predictive of most common linkage phases between RYR1 alleles and QTL alleles. Figure 4 shows that predicted QTL genotypic effects for pH-45 QTL (SSC6-74 cM) efficiently cluster the three RYR1 genotypes, while genotypes carrying the mutated Cys^615 ^allele are associated with negative genotypic values for the QTL affecting backfact thickness at the same position (average difference of QTL predicted effects between homozygous RYR1 genotypes of -1.56 mm or -0.45 SD). However, the dispersion of backfat QTL genotypic values within two of the three RYR1 genotypes also points to a segregation of backfat thickness QTL independently of the RYR1 mutation. The same analysis was performed for loin area QTL and showed an association between predicted QTL genotypic effect for loin area QTL (SSC6-98 cM) and RYR1 genotypes (average difference of QTL predicted effects between homozygous genotypes of +1.06 cm^2^ or +0.19 SD). Overall, these results suggest incomplete linkage disequilibrium in this population between the RYR1 Cys^615 ^allele and the QTL alleles for decreasing backfat levels and increasing loin muscle section area. However, these observations do not exclude a genuine causative effect of the RYR1 Cys^615 ^mutation on these carcass composition traits, independent of identified linked QTLs.

### Known causative mutations in QTL detection analyses

Segregation of known mutations was readily detected as highly significant QTLs at the genomic locations of the causative polymorphisms, using a population including a low frequency of mutant allele (*p *= 0.03-0.08) but using marker genotypes fully informative for causative mutations. This outcome was verified with pH-45 for RYR1 and glycolytic potential for PRKAG3, measurements of muscle physiology known to be directly affected by these gene functions. These results not only act as quality controls for record acquisition, genotyping procedures and statistical analysis but also illustrate the fact that the practical power to detect a major-effect haplotype in such an experiment is very high, provided genotypes are informative for QTL genotypes. QTL contribution to trait variance as reported here is a function of allele substitution effects, which were not estimated in this work (QTLs were modelled here as random effects, allowing fitting of any allelic series), and allele frequencies, which are population-dependent. The overall impact of RYR1 and PRKAG3 mutations in this dataset is obviously the result of very large allele substitution effects, as frequency of mutated alleles are known and relatively low (*p *= 0.03-0.08). Other QTLs detected in this work and showing a substantial impact on trait variance may result from smaller effects of more frequent alleles. Overall, these results point to the absence of any additional major-effect polymorphism affecting the same set of traits in this population, beyond the known RYR1 and PRKAG3 mutations. The very large effects of the RYR1 and PRKAG3 mutations can be seen as a consequence of the specific nature of these two polymorphisms, which are both non-conservative changes in the sequence of proteins encoded by these genes. However, even using these best possible conditions, we also observed that those major-effect mutations were detected as more typical significant or suggestive QTLs when analyzing less severely affected traits. Trait exposure to QTL effects appears to define opportunities for unambiguous QTL detection and ultimately QTL genotype prediction, as illustrated in Figure 4 for QTL genotypic values predicted from the pH-45 QTL analysis.

Whereas subtraction of PRKAG3 Gln^200 ^carriers from the control population effectively suppressed QTL detection on SSC15 for all affected traits (Figure 2), we uncovered a complex picture of genetic effects segregating on SSC6 (Figure 3). The RYR1 mutation carriers contribute to but cannot fully explain QTL segregation for backfat thickness, while exclusion or inclusion of RYR1 Cys^615 ^carriers does not interfere with QTLs detected for loin muscle section area. These results suggest that at least one extra polymorphism is segregating in this population in addition to the RYR1 mutation that affects backfat thickness. The RYR1-mutated animals contribute to the backfat QTL detection, but so do others. The detection of QTLs affecting loin muscle section area essentially takes no contribution from RYR1 mutation carriers, which might be interpreted as a fixation of either allele in families segregating for RYR1 alleles, or limited linkage disequilibrium of the two polymorphisms. The different contributions of RYR1 mutation carriers to backfat thickness and loin muscle area QTL detection on SSC6 as well as the different positions for most likely QTL locations (Figure 3, after exclusion of mutation carriers) further suggests that backfat thickness and loin muscle area QTLs could be driven by two independent polymorphisms in addition to the RYR1 mutation. QTLs affecting backfat thickness and loin muscle development located in overlapping SSC6 intervals had been reported previously in populations free of RYR1 mutation, further supporting this interpretation: a Duroc × Pietrain F2 population [10] and an Iberian × Landrace F2 population [22]. A similar observation was made using a different approach in an F2 population including carriers of the RYR1 Cys^615 ^mutation when analyzing QTL segregation on SSC6 after the correction of phenotypes for RYR1 mutation effects [24].

The RYR1 Cys^615 ^mutation was fixed in the Pietrain breed and showed a high frequency in heavily muscled pig breeds [1] intensively selected for low backfat thickness and increased muscle development. These breeding programs may have contributed to the building of SSC6 haplotypes combining the RYR1 mutation and QTL alleles reducing backfat thickness and increasing muscle development, detected as a combined effect [29,32].

Identification of the RYR1 gene as causative for MHS initially raised questions regarding the likelihood of physiological effects of the RYR1 Cys^615 ^mutation on fat deposition, and suggested neighbouring functional candidate genes (APOE, TGF-β1, LIPE) as causative for those effects [33]. A later study using a wild boar × domestic pig F2 population found an absence of QTL linked to the RYR1 mutation [34]. Our data provide evidence for such linked QTL and show that assessing pleiotropic *versus *linked effects in F2 populations is restricted to the availability of observable recombinations between causative polymorphisms in the population under study. Recombined haplotypes that we detected in this work are unlikely to be the product of the F2 intercross design we used, but were more likely sampled from the Pietrain parental population where the RYR1 mutation was segregating at a low frequency (*p *< 0.20). These recombined haplotypes may be more common in contemporary pig sire lines as a result of breeding practices limiting the frequency of the RYR1 mutated allele (owing to its detrimental effects on meat quality), while selecting for decreased backfat and increased muscle development. This observation also suggests that re-estimating the supposedly favourable effects of the RYR1 Cys^615 ^mutation on carcass composition within different purebred pig populations where it segregated for a sufficiently large number of generations would be more appropriate for a bias-free estimation of the mutation causative effects. Re-evaluating less favourable effects of this mutation on carcass composition traits would encourage pig breeders to eradicate the mutant allele in most contexts considering the unfavourable effects of the RYR1 Cys^615 ^mutation on meat quality [32].

The occurrence of multiple linked QTLs has been commonly described as underlying the segregation of genetic effects in the pig [26], mouse [35,36] and tomato [37]. Successive removal of major-effect loci has been proposed as an explicit strategy in yeast [38] to further characterize alternative sources of genetic variation from the same given chromosome segment. Our observations incidentally support the relevance of this strategy and illustrate the specific opportunities of incomplete linkage arising from recombination within outbred livestock populations.

## Conclusions

QTLs were identified for nearly all the meat quality and carcass composition traits analyzed, but more QTLs were detected for fat deposition, muscle development, and meat colour.

The interpretation of causative mutation control detection in addition to the detection of independent QTLs at neighbouring loci affecting the same traits suggests that part of the muscle development and backfat thickness effects that have been commonly associated with the RYR1 mutation [39,40] results from linkage with neighbouring QTLs affecting the same traits.

Four to twenty percent of phenotypic variance is explained by each of the newly detected QTLs in this study, which remains within the range of major-effect mutation effects on traits not directly affected by RYR1 and PRKAG3 mutations (Drip-L/RYR1 5%; LL-L*/PRKAG3 19%). Considering the large impact achieved by control of the RYR1 and PRKAG3 mutations on pork production worldwide, this direct comparison suggests that QTLs detected in this study may also drive a valuable proportion of the variation in pork production, thereby encouraging the identification of underlying polymorphisms.

Conversely, the very large effects of the RYR1 and PRKAG3 mutations on pH-45 and glycolytic potential demonstrate the efficiency of QTL analysis in discriminating QTL genotypes. This observation suggests that the identification of physiological traits directly affected by the segregation of detected QTL, such as the muscle glycogenic potential measurement [41] would be key in QTL genotype prediction and hence in the dissection of underlying causative polymorphisms. This conclusion calls for a systematic search of primarily affected traits as the most informative ones for predicting unknown QTL genotypes. Both candidate biochemical traits and measurements derived from functional genomic screens or metabolomic profiles are relevant here.

## Methods

### Experimental population

An F2 population of females and barrows was produced from an intercross between two production sire lines: FH016 (Pietrain type, France Hybrides, St-Jean-de-Braye, France) and FH019 (Synthetic line from Duroc, Hampshire and Large White founders, France Hybrides). Eighteen F1 males and 72 F1 females were produced from 16 FH016 F0 males and 25 FH019 F0 females. F2 animals (1,370) were produced by mating each of the 18 F1 boars to the same group of one to four full sib F1 sows for the production of one to three litters. All animals were raised on the same farm and were slaughtered in the same abattoir in 32 successive batches. The number of records collected on F2 animals ranged from 700 to 1,350 according to the trait (Table 1 and Additional File 3).

### Genetic markers

Genomic DNA was extracted from white blood cell samples from F0 and F1 animals using a salting-out procedure [42] and from piglet tails docked at birth for F2 animals using a DNeasy tissue extraction kit (QIAGEN, Courtabeuf, France). One hundred and seventy microsatellite loci spanning all autosomes with an average spacing of 17 cM were selected based on predicted information content of available markers in this population. Information content was assessed from the number of alleles observed and average heterozygosity when genotyping all F1 males (data not shown). Microsatellites were genotyped by PCR and electrophoretic sizing of the PCR products. The sequence of primers used in PCR followed the reference pig genetic maps ([43], NCBI Map viewer Sscrofa5) or are summarized in Additional File 4 for complementary markers. Amplification was carried out with fluorescently labelled primers (M13 tag-tailed primer and labelled M13 primer in the same PCR reaction, Dyes IRD700/IRD800, Sigma-Genosys, Lyon, France). PCR was followed by pooling markers across different size ranges and a denaturing polyacrylamide gel electrophoresis revealed on a LICOR 4200 automated sequencer (Li-COR Inc., NE). Microsatellite alleles were called by PCR fragment sizing and binning using Li-COR SAGA genotyping software and a custom set of co-migrating size standards (fluorescently labelled PCR products from a set of plasmid inserts). We separated F1 parents and F2 progeny segregating alleles on common gels to allow a direct match between parents and progeny alleles. All gels were manually reviewed and edited for the assessment of genotyping quality and genotype validation. In addition to microsatellite genotypes, the MC4R and PRKAG3 V199I RFLP polymorphisms were genotyped as described [44,45] and used as genetic markers. All F2, F1 and F0 animals were genotyped for all markers. We performed iterative Mendelian inheritance checks for each marker allele throughout the whole pedigree using the peeling algorithm implemented in the LOKI software package [46].

Multipoint linkage maps were constructed *ab initio *using CRIMAP 2.4 software [47]. Genetic maps used for QTL detection were built from all the genotypes available from this population including carriers of the RYR1 and PRKAG3 mutations using the CRIMAP build option. However, the order of the closest markers was fixed as inferred from physical maps [48,49] when a limited number of recombination events in this population did not allow proper assessment of marker order. The sex-averaged genetic maps used subsequently in IBD coefficient estimation and QTL detection analyses are reported in Additional File 4.

### RYR1 and PRKAG3 genotypes

All F2 animals were individually genotyped for the RYR1 R615C and PRKAG3 R200Q mutations using previously described RFLP tests [3,50]. These genotypes were used to define a population excluding carriers of either of the mutations we used for *de novo *QTL detection, while a control population included all the animals and was used for control detection of known mutations (mutant allele frequencies p RYR1 Cys^615 ^= 0.08; p PRKAG3 Gln^200 ^= 0.03). Additional File 3 lists the number of records per trait and per genotype analyzed in this control population including all animals or selectively removing mutation carriers. Mutation genotypes were subsequently included in the genetic marker genotypes used in QTL detection procedures, although they were only informative in the control population.

### Meat quality traits

Early pH fall in loin muscle was recorded as pH at 45 minutes in the loin (pH-45) and was measured from a solution of 1 g of longissimus lumborum muscle dispersed 45 minutes *post mortem *in 9 ml of a 5 mM sodium iodoacetate - 150 mM potassium chloride buffer. Ultimate meat pH was recorded in loin (longissimus lumborum muscle, pH-LL) and ham (semimembranosus muscle, pH-SM) 24 h *post mortem *on half-carcasses. Intramuscular fat content (IMF) was measured as percent lipid (lipid weight:meat weight) as determined by chloroform/methanol extraction from freeze-dried longissimus lumborum sampled 30 h *post mortem*. Glycolytic potential (Glyc-P) was measured on the same longissimus lumborum samples and calculated from the enzymatic quantification of glycogen, glucose-6P and lactate concentrations [41].

Loins were sliced 30 hours *post mortem*, and two 3-4 cm slices (326 g +/- 65 g; including 11^th^-12^th ^ribs) were collected. A first chop was used for loin colour measurement, raw meat shear force measurements and chemical analysis. The other chop was kept at 4°C for 48 h in a closed polyethylene plastic bag and then grill-cooked in a 240°C dry oven for 30 min. Drip loss was calculated as 100(1 − weight after storage/weight before storage). The same chop was weighed again after cooking and dripping and loin cooking yield was calculated as 100(weight after cooking/weight after storage). Raw meat and cooked loin instrumental tenderness were assessed by the measurement of shear force (Warner-Bratzler 60° cell, maximum strength over shearing of 1 cm diameter meat cylinders, average of ten repetitions). Loin lightness (CIE L*), redness (CIE a*), and yellowness (CIE b*) colorimetric parameters were recorded using a Minolta CR300 colorimeter (Konica Minolta Sensing Europe BV, Roissy, France). CIE L*, a* and b* colour measurements were acquired with the same equipment from deboned semimembranosus muscle 96 h *post mortem*. An in-depth account of procedures used for recording meat quality traits was reported previously [51,52].

### Carcass composition and growth traits

Backfat thickness and loin muscle cross-section area were recorded on live pigs at 105 kg bodyweight from echographic pictures of loin sections at the last rib level acquired using a 3.5 MHz linear transducer probe and an Aloka 500-V echograph (Aloka, Tokyo, Japan). Fat thickness and muscle depth were recorded from carcasses at slaughtering using the carcass grading system in use at the abattoir, Fat-O-Meter (SFK, Herlev, Denmark). Fat depth and muscle depth from this equipment are calculated from readings of an optical probe inserted through the skin, the back fat layers and the loin muscle and recording penetration depths at transitions between fat (white) and muscle (red) layers. Records were acquired at a dorsal (last rib) and a lumbar position, on a lateral line located 10 cm from the mid-line. A hand-operated optical probe (Introvison Ltd, Hitchin, UK) used on carcasses 24 h *post mortem *allowed a direct visual score of fat depths at similar locations. Fat depth was also recorded with a ruler on ham cuts directly above the humerus and on half-carcass mid-line cuts at a dorsal and lumbar position. The primal cut weights of ham without feet or loin but with bones were recorded at carcass cutting. Loin rib eye area was measured from digitized pictures of sliced chop. Individual birth weight was recorded on the first day of life and growth average daily gain was calculated as total weight gain during the *ad libitum *fed growth period (35 kg to 105 kg) divided by the number of days for this period. Elementary statistics for all traits analyzed along with abbreviations used and short definitions are presented in Table 1 for the F2 population excluding carriers of RYR1 and PRKAG3 mutations.

### Statistical analysis

The genetic variability of the population used in this study, where phenotypes were recorded in a single F2 generation, can be described as a segregation of F1 alleles and can be analyzed by interval mapping for QTL segregation within each of half-sib or full-sib F2 families [53]. However, parental haplotypes from these outbred populations cannot be treated as fixed in each parent population, and actual sizes of half-sib or full-sib families in our F2 population limits the detection power of within-family analyses or restrict analysis to the largest families. However, additional relationships exist in our pedigree beyond segregation within full-sib and half-sib families as F1 males and females used in different half-sib families were selected from the same F0 litters. Additionally, as all F1 females mated to the same F1 male were full sibs, relationships between F2 half sibs include sharing of identical F0 alleles transmitted by the different F1 females in addition to the segregation of paternal alleles. Use of sampling-based approaches associated with pedigree peeling makes it possible to infer sharing of identical-by-descent (IBD) founder alleles among all phenotyped (F2) animals [46].

We present results from a QTL detection analysis based on population-wide analyses of covariance between relatives associated with their IBD coefficients for each particular genomic position considered, as could be inferred from pedigree and inheritance of linked marker alleles. QTL detection was performed using a two-step variance component estimation method as proposed by George [54] and subsequently demonstrated in livestock [9,55]. Briefly, univariate mixed models of variance were fitted to trait observations under either an additive polygenic model (H0) or a QTL model (H1: additive polygenic effect and QTL effect) for each of the tested positions. Both variance analysis models included the same fixed effects describing slaughter or fattening batches (all traits) and sex (carcass composition traits, cooked loin shear force, glycolytic potential and intramuscular fat percentage). Carcass weight or live weight was used as a covariate in all carcass composition trait models. A covariate for chop weight was used in the loin cooking yield and cooked loin shear force models. An independent random effect was used to fit a common environment defined as birth in a common litter in the analysis of individual birth weight and average daily gain. Additive genetic effect was set up using a three-generation pedigree structure in addition to the phenotyped animals, and QTL effects were fitted using the IBD relationship matrix estimated for the given genome position. IBD relationship matrices were estimated using a reversible jump Monte Carlo Markov chain method every 4 cM along linkage groups using the software package LOKI 2.4.6 [46] and sampling over 20,000 iterations. No assumptions were made regarding IBD status of founder alleles originating from the same parental line, i.e. all IBD probabilities of founder alleles were set to 0, irrespectively of founder parental line origin. This was set to describe expected allele heterogeneity within these outbred parental lines, with known admixture history (one of the two parental lines being a synthetic line). Variance components were estimated using a residual maximum likelihood (REML) method with ASREML 2.0 software [56].

A QTL detection test was computed at each of the scanned positions using a likelihood ratio test (LRT) of −2(logikelihood-H1 − loglikelihood-H0).

It was suggested that this statistic followed a chi-squared distribution from 1 DF (for one specific position) to 2 DF when testing over a genetic interval [57]. The maximum LRT recorded for each scanned chromosome was compared with chromosome-wise thresholds obtained by simulation (see below) to assess QTL detection. The sum of estimated variance components as estimated from a polygenic additive model (H0) in a population excluding RYR1 and PRKAG3 mutation carriers was used to compute reference trait standard deviation in summary statistics of Table 1. The proportion of variance explained by each QTL effect was estimated as the ratio between the variance component associated with the IBD relationship matrix at most significant QTL position and the sum of variance components estimated from the same model (Additional File 5). In both cases, variance estimates refer to total phenotypic variance after correction for fixed effects and covariates used.

We checked total variance inflation or deflation between significant QTL models and polygenic (null hypothesis) models. Out of the significant and suggestive QTLs reported, we noted five cases where QTL model total variance deviated from additive model total variance by more than 2% (namely QTL for LL-a* (+5%), LL-b* (+3%) on SSC6, QTL for ADG on SSC1 (+3%) and QTL for F-FOM-B on SSC3 (−3%) and SSC5 (−4%)). In all other significant and suggestive QTL models, the sum of variance components was within 2% of the sum of variance components estimated under a polygenic model. We checked the sampling error of estimated QTL variance component, as estimated from the square root of the diagonal element of the average information matrix [56]. For all significant QTLs, this sampling error reported as a proportion of estimated variance component (component/sampling error ratio), ranged from 1.73 to 2.82 over 28 significant QTLs, with an average value of 2.07.

### Significance thresholds and confidence intervals

We inferred chromosome-wise significance thresholds from the distribution of QTL detection statistics observed on phenotypes simulated under the null hypothesis. We ran QTL detection using the same marker genotypes (IBD coefficients) and pedigree on sets of simulated phenotypes modelled from our pedigree structure as carrying polygenic additive variation only (*h*^2^ = 0.3). However, to save computing, we ran QTL autosome scans on a large number (5,000) of simulated phenotype datasets but using a smaller subset of animals (305 animals; 4 half-sib families). We also scanned QTLs on four representative test chromosomes (SSC1, SSC6, SSC15, and SSC18) over fewer (1,000) full-sized simulated datasets modelled from the population structure used for a typical meat quality trait record (SF-cook, *n *= 760). A summary of selected quantile values from the distribution of maximum LRT detected on these datasets simulated under the null hypothesis is tabulated for each chromosome in Additional File 6. Chromosome-wise significance thresholds were found to vary to some extent according to linkage group size, but were not influenced by dataset size (Additional File 6), consistent with the expected distribution of nominal test statistic. Empirical simulation-based chromosome-wise thresholds were found to be close to corresponding quantiles from a χ^2^_2DF _distribution, the upper bound theoretical estimate proposed for a genetic interval [57] (1% quantiles were found ranging from 7.7 to 9.5 over the 18 autosomes, compared with a 1% quantile from χ^2^_2DF _of 9.21).

Simulations were performed only once and quantiles drawn from the null hypothesis LRT distribution of the largest set of simulations (5,000) using small datasets for each chromosome were used as chromosome-specific chromosome-wise significance thresholds throughout all traits analyzed in this work, irrespective of the actual number of records in each analysis. We considered only maximum QTL detection statistics across all positions along each linkage group against these chromosome-wise thresholds without considering any secondary peaks. Genome-wise significance levels were adjusted using a Bonferroni correction as suggested previously [5], and QTLs are reported as significant for a 5% genome-wise significance level (equivalent to a 0.285% chromosome-wise level for independent scanning of 18 autosomes per genome). QTLs detected with an LRT above 1% or 5% chromosome-wise threshold but below the 5% genome-wise threshold are reported as suggestive QTLs. A 1% genome-wise threshold is proposed to support interpretation of the most significant QTL detection tests in figures and tables as drawn from a χ^2^_2DF _distribution and using the same Bonferroni correction for the testing of 18 chromosomes (1% genome-wise threshold corresponding to a 0.058% chromosome-wise threshold).

Confidence intervals are proposed on the basis of a -1 LOD drop-off and are reported as 95% confidence interval map segments where the loglikelihood of the QTL model is higher than loglikelihood (max) − ln(10) [58]. Predicted QTL genotypic effects were obtained from solutions of mixed model equations for QTL models fitted at most significant chromosome position defined by maximum LRT.

### Comparison of results with published QTLs

We identified parallels between significant QTLs detected in this study and QTLs reported in the literature for similar traits by searching the online directory of published QTLs, the pig section of the AnimalQTLdb release 12 [59]. However, in most cases, the very large number of QTLs (5,986) described in pigs, and the very large confidence intervals associated with F2 and backcross studies, prevent formal identity confirmations with our detected QTLs.

## Competing interests

The authors declare that they have no competing interests.

## Authors' contributions

PC coordinated the study, analyzed data and drafted the paper. JP and JG carried out genotyping and phenotype measurements. JP reviewed all genotypes and checked marker Mendelian inheritance. MD and FH participated in meat composition measurements and QTL analyses. NI carried out screening of informative markers. DM assisted in marker selection, genotyping protocols and study design. PLR conceived the study and reviewed the QTL analyses. All the authors read and approved the final manuscript.

## Supplementary Material

Additional file 1 **Suggestive QTLs detected for meat quality, carcass composition and growth traits**. Suggestive QTLs detected in the F2 population excluding carriers of either RYR1 or PRKAG3 mutations, at 5% and 1% chromosome-wise significance levels. Click here for file

Additional file 2 **QTL detection LRT profiles for carcass composition traits on chromosomes SSC5, SSC11 and SSC13**. LRT profiles for groups of carcass composition traits where significant and suggestive QTL were detected at neighbouring locations. Click here for file

Additional file 3 **Detection of RYR1 and PRKAG3 mutations in the control population**. The numbers of records for each genotype are tabulated with corresponding mutation frequency for a selection of traits. QTL detection results including or excluding mutation carriers are summarized for the RYR1 mutation on SSC6 and the PRKAG3 mutation on SSC15. Click here for file

Additional file 4 **Sex-averaged genetic maps**. Genetic map of 18 pig autosomes used for QTL detection including the list of marker names and positions for all genetic markers used. Individual marker positions are expressed in Haldane mapping distance function from the first marker in each linkage group. Primer sequence information for complementary genetic markers not available in reference genetic maps is included. Click here for file

Additional file 5 **Estimated variance components in analyses fitted at all significant and suggestive QTL positions**. All variance components are reported in trait units; genetic components (additive and QTL effects) are reported as percentage of total variance. Click here for file

Additional file 6 **Chromosome-wise significance thresholds**. Chromosome-wise thresholds as quantiles of the distribution of QTL detection statistical test used when applied to phenotypes simulated under the null hypothesis (polygenic additive model, H0: *h*^2^ = 30%). Click here for file
