# Supplementary material for: Joint analysis of quantitative trait loci and major-effect causative mutations affecting meat quality and carcass composition traits in pigs
Source: BMC Genet. 2011 Aug 29;12:76. doi: 10.1186/1471-2156-12-76 (PMC3175459; doi:10.1186/1471-2156-12-76)
Supplement: Additional file 1 — Suggestive QTLs detected for meat quality, carcass composition and growth traits. Suggestive QTLs detected in the F2 population excluding carriers of either RYR1 or PRKAG3 mutations, at 5% and 1% chromosome-wise significance levels. [file 1471-2156-12-76-S1.PDF]

**Suggestive QTL detected for meat quality, carcass composition and growth traits**

QTL detected in F2 population excluding carriers of either RYR1 or PRKAG3 mutations.

[1]Position of most significant QTL detection on genetic map in cM (1-LOD drop-off confidence interval). [2]LRT significance levels: $^{+}$ 5% chromosome-wise; $^{++}$ 1% chromosome-wise;. [3]Percentage of phenotypic variance associated with QTL effect.

| SSC | Trait | Max (95% CI)[1] | LRT[2] | QTL v.[3] | Flanking markers | |
|---|---|---|---|---|---|---|
| 1 | LMA-US | 24 (12-44) | 11.1 $^{++}$ | 4.8% | Sw552 | Sw1515 |
| | F-HCr-B | 61 (45-81) | 7.0 $^{+}$ | 5.1% | Sw1515 | Sw1851 |
| | F-FOM-B | 64 (44-456) | 8.5 $^{+}$ | 5.5% | Sw1851 | CGA |
| | F-HCr-L | 65 (41-89) | 7.5 $^{+}$ | 4.2% | Sw1851 | CGA |
| | F-oP-L | 73 (45-173) | 6.6 $^{+}$ | 5.3% | Sw1851 | CGA |
| | LL-pH | 106 (96-116) | 11.0 $^{++}$ | 7.9% | MC4R | FH2510 |
| | Cook-Y | 108 (88-124) | 6.6 $^{+}$ | 13.9% | MC4R | FH2510 |
| | LMA-C | 120 (104-164) | 6.6 $^{+}$ | 4.9% | FH2510 | Sw501 |
| | LOIN-W | 120 (36-252) | 6.6 $^{+}$ | 4.6% | FH2510 | Sw501 |
| | F-US | 180 (128-212) | 9.3 $^{+}$ | 8.3% | FH1231 | MCS455 |
| | Ham-W | 204 (196-212) | 9.1 $^{+}$ | 7.1% | MCS455 | Sw1301 |
| 2 | SF-Cook | 32 (0-64) | 8.4 $^{+}$ | 14.9% | MCS141 | S0141 |
| 3 | SM-pH | 1 (0-25) | 11 $^{++}$ | 8.6% | APR22 | APR22 |
| | F-FOM-B | 9 (0-32) | 6.9 $^{+}$ | 9.7% | APR22 | Sw72 |
| | F-Ham | 13 (0-41) | 6.2 $^{+}$ | 11.0% | APR22 | Sw72 |
| | LMA-US | 84 (0-128) | 6.0 $^{+}$ | 4.5% | S0032 | Sw271 |
| | M-FOM-B | 89 (65-109) | 8.6 $^{+}$ | 6.0% | S0032 | Sw271 |
| | LL-b* | 160 (136-176) | 9.9 $^{++}$ | 8.3% | Sw717 | Sw2532 |
| 4 | F-oP-B | 28 (0-52) | 5.8 $^{+}$ | 5.6% | Sw480 | Sw2049 |
| | pH-45 | 40 (0-100) | 6.8 $^{+}$ | 8.3% | Sw2547 | Sw2409 |
| | SM-L* | 80 (60-96) | 6.1 $^{+}$ | 10.1% | Sw45 | S0217 |
| | SF-Cook | 89 (57-113) | 5.7 $^{+}$ | 9.9% | S0217 | S0073 |
| | LMA-US | 116 (84-148) | 7.1 $^{+}$ | 6.1% | S0073 | Sw524 |
| | IMF | 120 (104-140) | 8.5 $^{+}$ | 10.8% | Sw524 | Sw445 |
| | LL-L* | 124 (104-136) | 6.1 $^{+}$ | 7.6% | Sw524 | Sw445 |
| 5 | Glyc-P | 144 (88-168) | 9.5 $^{++}$ | 9.0% | FH6061 | FH1701 |
| | M-FOM-L | 161 (121-181) | 6.7 $^{+}$ | 4.7% | S0005 | Sw1468 |
| | pH-LL | 164 (60-220) | 5.8 $^{+}$ | 5.0% | S0005 | Sw1468 |
| | SF-raw | 176 (56-220) | 5.8 $^{+}$ | 6.2% | Sw1468 | IGF1 |
| | F-HCr-B | 221 (197-245) | 6.6 $^{+}$ | 8.7% | IGF1 | Swr378 |
| 6 | LL-pH | 44 (16-72) | 8.2 $^{+}$ | 6.2% | Sw1038 | Sw1067 |
| | LL-L* | 60 (12-80) | 8.5 $^{+}$ | 7.2% | Sw1038 | Sw1067 |
| | F-US | 64 (48-88) | 9.2 $^{++}$ | 5.7% | Sw1038 | Sw1067 |
| | F-FOM-B | 64 (32-80) | 7.3 $^{+}$ | 4.9% | Sw1038 | Sw1067 |
| | IMF | 80 (56-108) | 8.7 $^{+}$ | 5.6% | Sw2521 | Sw122 |
| | Loin-W | 96 (68-112) | 7.8 $^{+}$ | 4.0% | Sw122 | Sw71 |
| | ADG | 101 (89-125) | 8.0 $^{+}$ | 6.0% | Sw71 | S0228 |

| | | | | | | |
|---|---|---|---|---|---|---|
| **7** | Birth-W | 40 (20-54) | 9.5$^{++}$ | 5.5% | Sw2155 | LRA1 |
| | IMF | 48 (28-92) | 9.4$^{++}$ | 6.9% | Sw2155 | LRA1 |
| | ADG | 49 (29-85) | 6.6$^{+}$ | 4.5% | Sw2155 | LRA1 |
| | Ham-W | 56 (36-84) | 6.5$^{+}$ | 5.0% | TNFb | Sw1856 |
| | F-FOM-B | 88 (72-104) | 7.9$^{+}$ | 5.7% | Sw1856 | Sw1614 |
| | M-FOM-L | 109 (97-153) | 8.2$^{+}$ | 10.3% | Sw1614 | Sw632 |
| **8** | pH-45 | 16 (0-32) | 5.9$^{+}$ | 8.7% | KS148 | S0098 |
| | LMA-C | 92 (48-124) | 9.6$^{++}$ | 7.9% | Sw205 | FH1089 |
| **9** | SM-pH | 153 (117-173) | 8.2$^{++}$ | 9.7% | Sw174 | Sw1349 |
| | M-FOM-L | 169 (141-173) | 7.6$^{+}$ | 9.2% | Sw174 | Sw1349 |
| **11** | F-FOM-L | 141 (113-149) | 6.4$^{+}$ | 12.3% | S0230 | Sw2413 |
| **12** | pH-45 | 20 (0-52) | 8.8$^{++}$ | 7.0% | FH1993 | Sw957 |
| | F-HCr-B | 25 (1-65) | 5.3$^{+}$ | 8.9% | FH1993 | Sw957 |
| **13** | pH-45 | 36 (4-68) | 6.6$^{+}$ | 11.9% | Swr1941 | Sw344 |
| | M-FOM-B | 77 (65-93) | 11.0$^{++}$ | 5.0% | Sw344 | Sw882 |
| | M-FOM-L | 77 (61-93) | 10.3$^{++}$ | 5.6% | Sw344 | Sw882 |
| | ADG | 85 (33-157) | 5.9$^{+}$ | 3.9% | Sw882 | Sw129 |
| | LMA-US | 88 (28-144) | 10.4$^{++}$ | 7.5% | Sw882 | Sw129 |
| | F-oP-L | 189 (153-189) | 6.6$^{+}$ | 12.7% | Sw1056 | S0291 |
| **14** | SF-cook | 33 (1-57) | 6.4$^{+}$ | 6.6% | Sw245 | Sw6 |
| | M-FOM-L | 33 (17-49) | 5.9$^{+}$ | 6.5% | Sw245 | Sw6 |
| | LMA-C | 56 (28-72) | 9.4$^{++}$ | 6.8% | Sw104 | Swr925 |
| **15** | ADG | 17 (0-33) | 8.9$^{+}$ | 8.1% | S0355 | S0148 |
| | SF-raw | 45 (20-72) | 8.5$^{+}$ | 11.9% | FH1710 | Sw1989 |
| | M-FOM-L | 141 (69-141) | 5.7$^{+}$ | 12.1% | FH1572 | SW1339 |
| **16** | F-HCr-B | 1 (1-25) | 4.8$^{+}$ | 4.3% | FH1853 | FH2314 |
| | F-oP-L | 1 (1-25) | 5.5$^{+}$ | 4.6% | FH1853 | FH2314 |
| | SM-a* | 8 (0-24) | 7.6$^{+}$ | 11.0% | Sw1035 | Sw1809 |
| | F-oP-B | 8 (0-36) | 5.6$^{+}$ | 4.0% | Sw1035 | Sw1809 |
| | F-Ham | 9 (0-37) | 8.5$^{++}$ | 6.5% | Sw1835 | Sw1809 |
| | SM-b* | 12 (0-36) | 6.2$^{+}$ | 8.8% | Sw1035 | Sw1809 |
| | Cook-Y | 20 (0-72) | 8.2$^{++}$ | 8.4% | Sw1035 | Sw1809 |
| | F-us | 36 (8-60) | 6.6$^{+}$ | 4.3% | Sw1809 | S0026 |
| | SF-cook | 49 (13-81) | 5.2 | 5.4% | S0026 | Swr2480 |
| | F-FOM-L | 81 (61-85) | 7.9$^{+}$ | 8.6% | Swr2480 | S0105 |
| **17** | SM-a* | 48 (28-64) | 8.5$^{+}$ | 7.2% | Sw2441 | S0292 |
| **18** | IMF | 20 (0-40) | 7.5$^{+}$ | 13.5% | Sw1023 | Sw787 |
| | F-oP-B | 28 (4-56) | 7.4$^{+}$ | 8.3% | Sw1023 | Sw787 |
| | LMA-C | 40 (16-76) | 4.9$^{+}$ | 6.1% | Sw787 | Sw1682 |
| | F-oP-L | 73 (17-81) | 5.7$^{+}$ | 7.2% | Sw1682 | FH1006 |
| | LL-pH | 80 (44-80) | 4.7$^{+}$ | 3.7% | Sw1682 | FH1006 |
| | F-HCr-B | 81 (1-81) | 5.4$^{+}$ | 8.6% | Sw1682 | FH1006 |
