# Supplementary material for: Joint analysis of quantitative trait loci and major-effect causative mutations affecting meat quality and carcass composition traits in pigs
Source: BMC Genet. 2011 Aug 29;12:76. doi: 10.1186/1471-2156-12-76 (PMC3175459; doi:10.1186/1471-2156-12-76)

<u>1/ Detection of QTL for five fat deposition traits on SSC5</u>

LRT profiles for QTL detection on SSC5 (Genetic markers used are represented with filled blue triangles on top of x-axis)

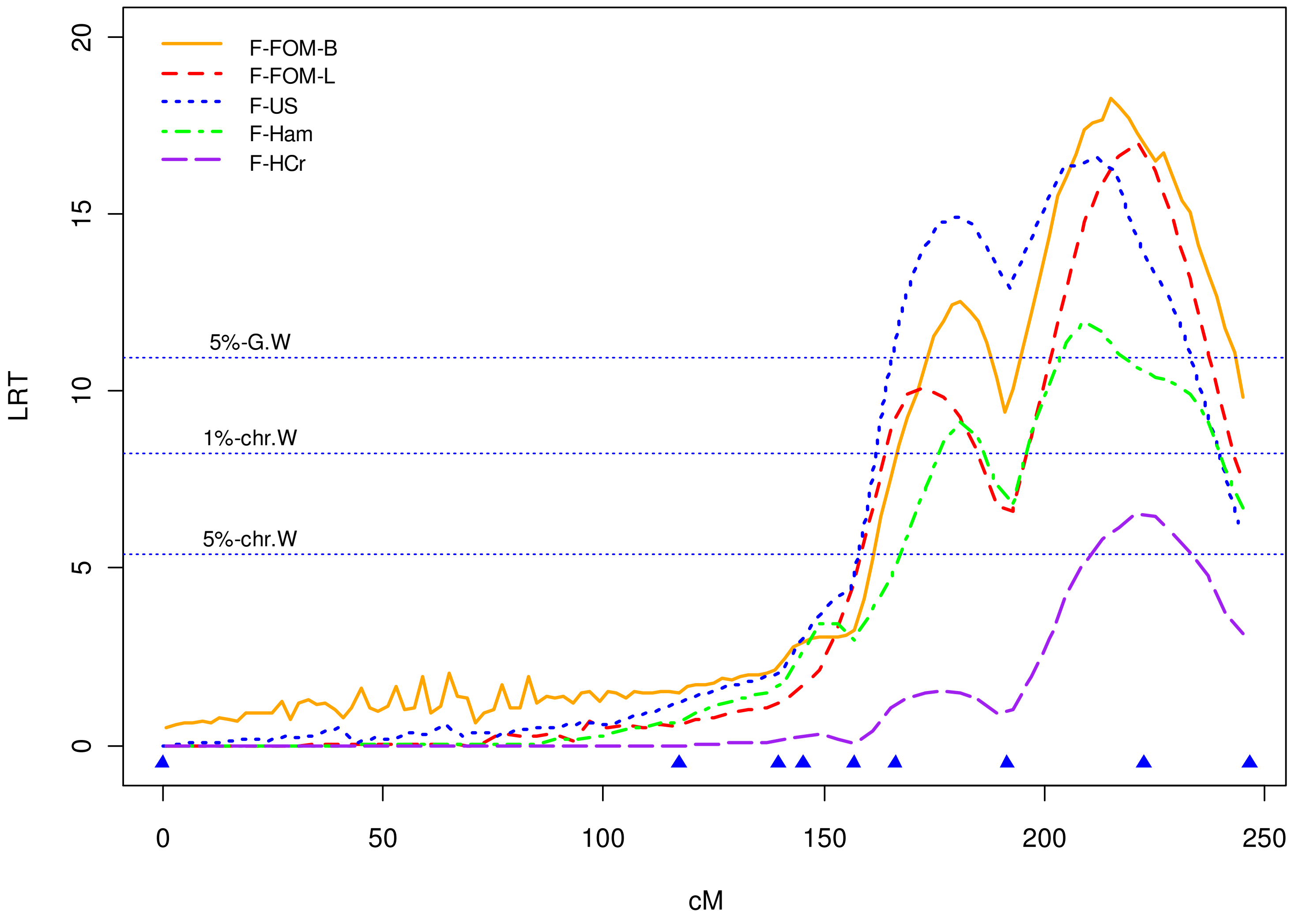

2/ Detection of QTL for four fat deposition traits on SSC11

LRT profiles for QTL detection on SSC11 (Genetic markers used are represented with filled blue triangles on top of x-axis)

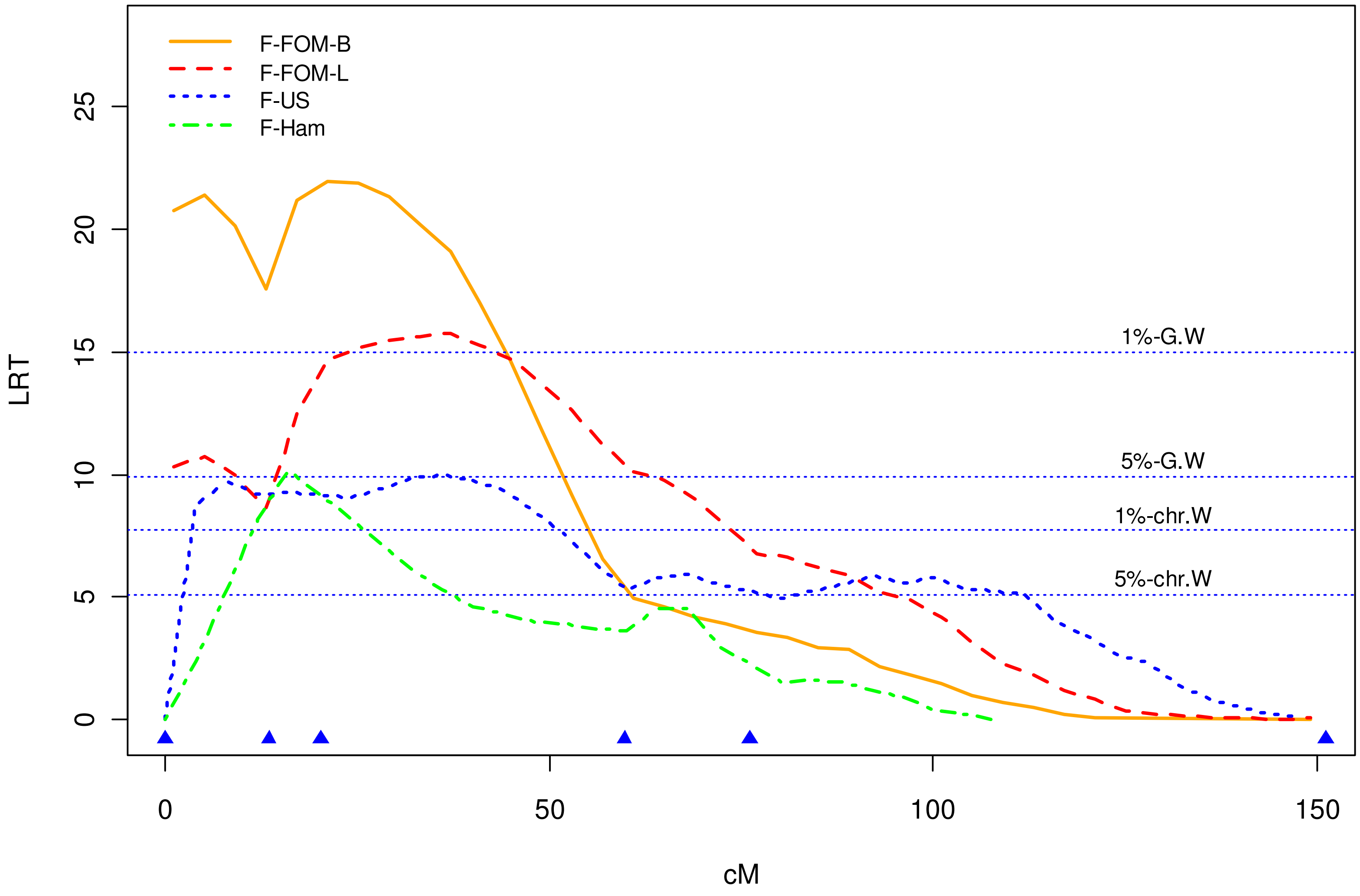

<u>3/ Detection of QTL for four muscle development traits on SSC13</u>

LRT profiles for QTL detection on SSC13 (Genetic markers used are represented with filled blue triangles on top of x-axis)

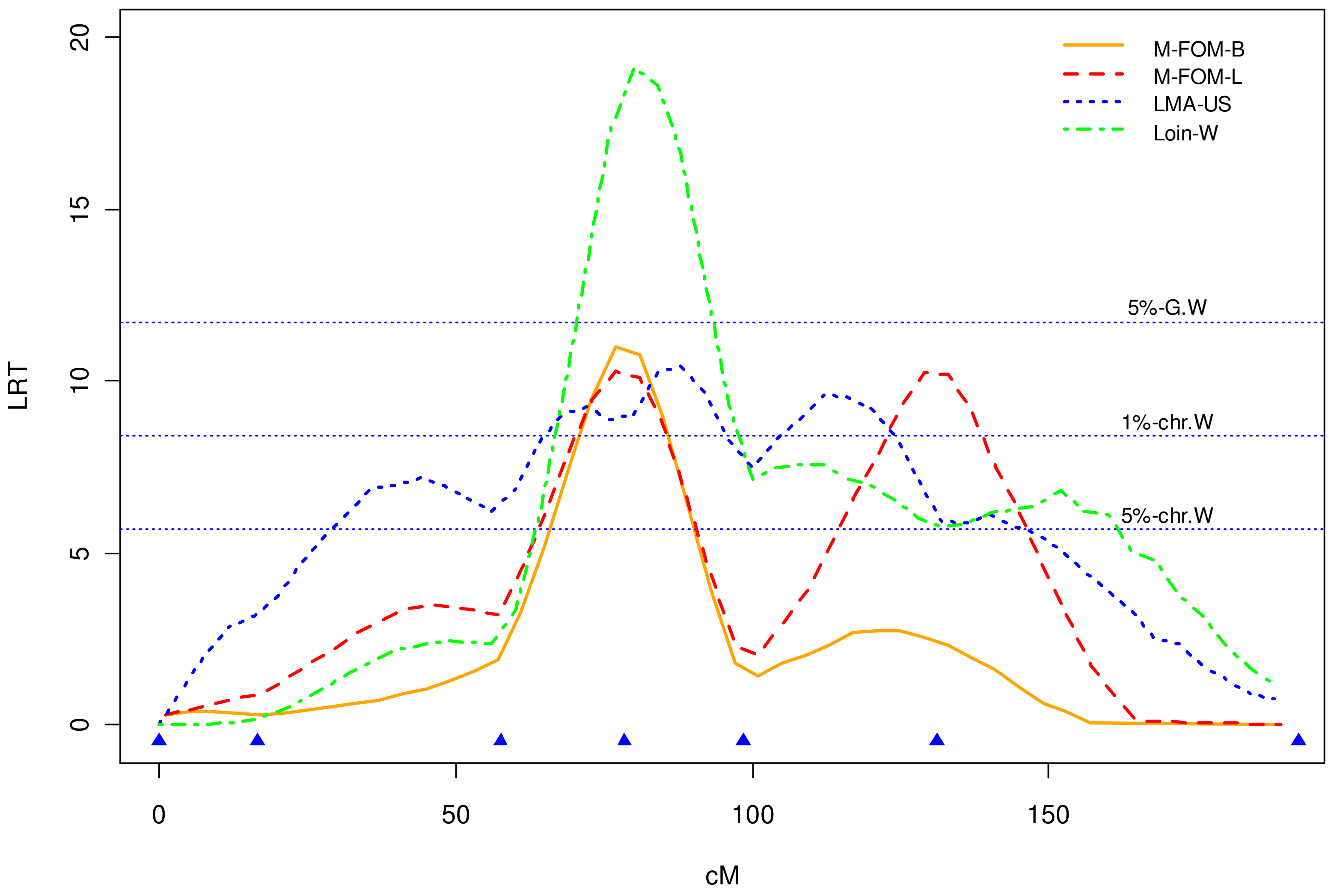

Supplement: Additional file 2 — QTL detection LRT profiles for carcass composition traits on chromosomes SSC5, SSC11 and SSC13. LRT profiles for groups of carcass composition traits where significant and suggestive QTL were detected at neighbouring locations. [file 1471-2156-12-76-S2.PDF]
