# Supplementary material for: Joint analysis of quantitative trait loci and major-effect causative mutations affecting meat quality and carcass composition traits in pigs
Source: BMC Genet. 2011 Aug 29;12:76. doi: 10.1186/1471-2156-12-76 (PMC3175459; doi:10.1186/1471-2156-12-76)
Supplement: Additional file 3 — Detection of RYR1 and PRKAG3 mutations in the control population. The numbers of records for each genotype are tabulated with corresponding mutation frequency for a selection of traits. QTL detection results including or excluding mutation carriers are summarized for the RYR1 mutation on SSC6 and the PRKAG3 mutation on SSC15. [file 1471-2156-12-76-S3.PDF]

QTL detection in Control population

QTL detection on SSC6

RYR1 position on genetic map is 73 cM

	RYR1 genotypes				***Including RYR1 Cys615, p > 0***				***Excluding RYR1 Cys615, p = 0***			
	**Cys/Cys**	**Cys/Arg**	**Arg/Arg**	**p(Cys615)**	**LRT**	**QTL v.**	**n**	**pos.**	**LRT**	**QTL v.**	**n**	**pos.**
**pH-45**	17	133	913	7.86%	*195.1 ***	34%	1063	74 cM	*1.4*	n.s.	913	n.s.
**Drip-L**	14	119	807	7.82%	*8.9 ++*	5%	940	74 cM	*2.2*	n.s.	807	n.s.
**F-op-B**	16	125	841	7.99%	*24.9 ***	9%	982	74 cM	***12.9 ****	**8%**	**841**	**64 cM**
**F-US**	30	179	1208	8.43%	*42.4 ***	8%	1417	74 cM	***13.1 ****	**6%**	**1208**	**64 cM**
**LMA-US**	30	179	1210	8.42%	*11.4 **	5%	1419	98 cM	***9.7 ++***	**5%**	**1210**	**98 cM**
**Loin-W**	17	155	1158	7.11%	*8.2 +*	4%	1330	74 cM	***7.8 +***	**4%**	**1158**	**96 cM**

QTL detection on SSC15

PRKAG3 position on genetic map is 95 cM

	PRKGA3 genotypes				***Including PRKAG3 Gln200, p > 0***				***Excluding PRKAG3 Gln200, p = 0***			
	**Gln/Gln**	**Gln/Arg**	**Arg/Arg**	**p(Gln200)**	**LRT**	**QTL v.**	**n**	**pos.**	**LRT**	**QTL v.**	**n**	**pos.**
**PotGlyc**	0	60	943	2.99%	*287.4 ***	*53%*	1003	94 cM	*3.5*	n.s.	943	n.s.
**pH-LL**	6	157	1181	6.29%	*160.0 ***	*36%*	1344	94 cM	*2.8*	n.s.	1181	n.s.
**pH-SM**	6	157	1182	6.28%	*111.2 ***	*25%*	1345	94 cM	*2.6*	n.s.	1182	n.s.
**LL-L***	3	80	984	4.03%	*25.0 ***	*19%*	1067	96 cM	*0.0*	n.s.	984	n.s.
**LL-a***	3	80	984	4.03%	*29.2 ***	*17%*	1067	94 cM	*0.4*	n.s.	984	n.s.
**LL-b***	3	80	984	4.03%	*8.9 +*	*9%*	1067	94 cM	*0.1*	n.s.	984	n.s.
**SF-cook**	3	79	887	4.39%	*4.2*	n.s.	969	n.s.	*0.4*	n.s.	887	n.s.
**pH-45**	4	67	983	3.56%	*2.4*	n.s.	1054	n.s.	*3.8*	n.s.	983	n.s.
**IMF**	0	54	842	3.01%	*0.0*	n.s.	896	n.s.	*0.0*	n.s.	842	n.s.

LRT significance levels: + 5% chromosome-wise; ++ 1% chromosome-wise; * 5% genome-wise; ** 1% genome-wise.
QTL v.: Proportion of phenotypic variance explained by QTL effect in QTL model
n.s.: Non-significant and non-suggestive QTL detection over whole chromosome for this trait and this population
