# Supplementary material for: Joint analysis of quantitative trait loci and major-effect causative mutations affecting meat quality and carcass composition traits in pigs
Source: BMC Genet. 2011 Aug 29;12:76. doi: 10.1186/1471-2156-12-76 (PMC3175459; doi:10.1186/1471-2156-12-76)
Supplement: Additional file 4 — Sex-averaged genetic maps. Genetic map of 18 pig autosomes used for QTL detection including the list of marker names and positions for all genetic markers used. Individual marker positions are expressed in Haldane mapping distance function from the first marker in each linkage group. Primer sequence information for complementary genetic markers not available in reference genetic maps is included. [file 1471-2156-12-76-S4.PDF]

Genetic maps of 18 pig autosomes used in QTL detection

SSC1

Sw1514	-
Sw552	*15.3*
Sw1515	*24.99*
Sw1851	*63.04*
CGA	*77.08*
S0122	*87.72*
Sw2185	*94.86*
MC4R	*104.2*
FH2510	*116.4*
Sw501	*121.65*
S0155	*125.81*
S0302	*142.64*
FH1231	*161.29*
MCS455	*195.25*
Sw1301	*205.41*
Sw2512	*213.47*

SSC2

Sw2443	-
Swc9	*1.7*
MCS141	*9.2*
S0141	*50.51*
Sw1450	*51.52*
Sw461	*65.8*
Sw1564	*82.39*
S0226	*101.44*
Sw1695	*110.19*
S0368	*113.17*
FH2152	*145.35*
Swr308	*180.97*

SSC3

APR22	-
Sw72	*48.14*
FH1589	*59.85*
Sw2527	*68.84*
S0032	*77.82*
Sw271	*98.19*
S0216	*117.23*
Sw2408	*134.84*
S0002	*143.48*
FH1085	*156.78*
Sw717	*159.66*
Sw2532	*180.43*

SSC4

Sw480	-
Sw2049	*32.03*
Sw2547	*37.95*
Sw2409	*53.75*
Sw45	*71.75*
S0217	*85.8*
S0073	*95.6*
Sw524	*117.18*
Sw445	*125.47*
S0097	*149.23*
Sw1461	*152.96*

SSC5

Sj024	-
Swr453	*117.01*
FH6061	*139.54*
FH1701	*145.12*
S0005	*156.71*
Sw1468	*166.05*
IGF1	*191.47*
Swr378	*222.48*
Sw967	*246.38*

SSC6

Sw2406	-
Sw1038	*41.3*
Sw1067	*68.7*
RYR1	*73.4*
Sw2521	*76.06*
Sw122	*84.01*
Sw71	*99.31*
S0228	*111.27*
S0121	*123.35*
LEPR-CA	*128.49*
Sw322	*168.07*
Sw1328	*173.76*

SSC7

Sw1873	-
FH1042	*3.52*
Sw2155	*31.92*
LRA1	*51.35*
TNFb	*54.44*
Sw1856	*60.47*
Sw1614	*91.04*
Sw632	*119.58*
S0101	*157.63*
FH2367	*180.42*
Sw1303	*195.1*

SSC8

KS148	-
S0098	*16.57*
KS195	*55.85*
Sw205	*82.83*
FH1089	*95.88*
Sw1679	*100.8*
S0225	*125.39*
Sw1671	*142.61*
Sw61	*155.54*
KS140	*185.38*

SSC9

Sw21	-
Sw911	*30.57*
Sw1434	*75.49*
Sw1677	*81.63*
Sw1435	*106.36*
Sw2093	*110.19*
Sw174	*134.23*
Sw1349	*175.06*

SSC10

S0038	-
Sw767	*22.8*
S0351	*55.13*
S0070	*77.65*
Sw1103	*107.35*
Swr67	*160.56*

SSC11

S0385	-
Sw1460	*13.43*
Sw2008	*20.12*
Sw151	*59.71*
S0230	*76.03*
Sw2413	*151.08*

SSC12

Sw2490	-
FH1993	*7.03*
Sw957	*43.25*
Sw1350	*72.95*
Sw37	*82.75*
S0090	*100.23*
S0106	*124.4*
Swr1021	*141.62*

SSC13

S0282	-
Swr1941	*16.57*
Sw344	*57.56*
Sw882	*78.46*
Sw129	*98.56*
Sw1056	*131.19*
S0291	*192.13*

SSC14

Sw857	-
Sw1631	*3.62*
Sw2038	*13.55*
Sw1125	*22.53*
S0063	*30.59*
Sw540	*31.6*
Sw245	*32.21*
Sw6	*38.68*
Sw104	*43.05*
Swr925	*58.99*
FH5058	*88.39*
FH5078	*96.8*
Sw2515	*130.91*

SSC15

S0355	-
S0148	*23.9*
FH1710	*35.37*
Sw1989	*58.44*
Swr1002	*74.38*
PRKAG3	*95.33*
Sw936	*100.69*
FH5172	*105.94*
Sw1339	*145.99*

SSC16

FH1853	-
FH2314	*3.09*
Sw1035	*5.03*
Sw1809	*26.6*
S0026	*43.05*
Swr2480	*53.33*
S0105	*88.49*

SSC17

SwN335	-
Swr1004	*16.06*
Sw24	*24.82*
S0296	*35.09*
Sw2142	*43.85*
Sw2441	*45.47*
S0292	*64.65*
FH2100	*81.35*
Sj063	*89.52*

SSC18

SY2	-
Sw1023	*8.52*
Sw787	*39.09*
Sw1682	*54.39*
FH1006	*83.8*

Note: Genetic distances are expressed using Haldane distance function

Marker name	PCR forward primer sequence (5'>3')	PCR reverse primer sequence (5'>3')
FH1006	ATACCCGCTAAGAAACTGCATC	TGATTCCTGACCTTGCTCCA
FH1042	CACCTAAGGCAATGGACACCT	CAGGGATGGAACCTGCATAC
FH1085	TGGGCTTGATGGTTGAAGA	GAGGATTGTAAAGATAAGGCAGAG
FH1089	TGCCACTTTAATAATGGGATAGC	GCAAGACAATATCCCAAAGTGTAG
FH1231	GGAGAGAATCAAAGGGAAACAGT	TGCTCCTGTGGTCAGAGTTTG
FH1589	CACAGGAGCAGCCCTAGATAA	AGGAATTGGGTAGAAGTTCGTG
FH1701	CTTGTCAGCCTGTAGCAAATATC	AAGGAAACCAATAGTATGGAGATG
FH1710	GTGCCTCCCACACTCCTCT	TAGGATTTCAACGTGAGCATTT
FH1853	ACCTCATTGTACCGGATTTGTT	CACAGCCTAAGTGGATGAATGT
FH1993	AATTCACAACAGAGCTCAAGGA	TGCGGAGTCTTACAGGAAATAA
FH2100	CACGAAATCCTCATAGCCAAG	TCAGACTTTGAACTCCAGAACTGT
FH2152	CTCATATGTCTAGTCACTTATGATGGA	CTGCTAGCTTTGGGCTTGC
FH2314	GTTCCAGTCTGCCCTTTCTTAC	CTGTCCAATAGGCCAGCAAT
FH2367	TGGTTGAATGGGTCAAGAAA	AACCTACTCACCTCAAGGTCCA
FH2510	CCATGGGTGCATCCCTAAA	TTCCTAATGACTTGTGGTGTTGA
FH5058	TGGAGCCACATATTCCTTCTG	CAAACCTATACCGCAACAGTGA
FH5078	CCTGACTTAGTGTCCATGAGGA	CCCGACTCTAGAATCAAACTGC
FH5172	ACTCCTGGCCTTGCTCAGT	TGGCGACATGTTCACTCAT
FH6061	CATAATTGCCCTTTGGGATTT	CTGAACCTCAGTTTCTCCAAGC
LRA1	CAGGGAAGGAACCCACATC	CCTGTGTTTCTATGGCTGTGC
MCS141	GGCTCTCTGTTCCCTGATTCC	CAGCTACGAGGTCCAGTCTTCC
MCS455	TCTGAAGATTTGGTGGCATAC	CAACACCCTGAAGCTGGAA
