# Supplementary material for: Joint analysis of quantitative trait loci and major-effect causative mutations affecting meat quality and carcass composition traits in pigs
Source: BMC Genet. 2011 Aug 29;12:76. doi: 10.1186/1471-2156-12-76 (PMC3175459; doi:10.1186/1471-2156-12-76)
Supplement: Additional file 5 — Estimated variance components in analyses fitted at all significant and suggestive QTL positions. All variance components are reported in trait units; genetic components (additive and QTL effects) are reported as percentage of total variance. [file 1471-2156-12-76-S5.PDF]

Variance Components, as estimated in analysis models fitted at all significant and suggestive QTL positions

All variance components are reported in traits units, genetic components are reported as percentage of total variance
QTL effect is reported as percentage of the sum of genetic components
QTL detection in F2 population excluding carriers of either RYR1 or PRKAG3 mutations
(1) Position of most significant QTL detection; (2) Likelihood Ratio Test; (3) chrW chromosome-wise, GW genome-wise
Abbreviations: Var. Variance; Add. Additive; Gen. Genetic; Com. Env. Common Environnement;na not applicable

SSC	Trait	position cM (1)	LRT (2)	Significance level (3)	QTL (IBD) Variance	Additive Variance	Com. Env. Variance	Residual Variance	Add. Var. %Var. Tot.	QTL Var. %Var. Tot.	QTL Var. %Var. Gen.
1	LMA-US	24	11.14	1%-chrW	1.42E+00	1.51E+01	na	1.33E+01	50.7%	4.8%	8.6%
1	F-HCr-B	61	6.98	5%-chrW	4.96E-01	2.61E+00	na	6.65E+00	26.7%	5.1%	16.0%
1	F-FOM-B	64	8.46	5%-chrW	4.22E-01	2.87E+00	na	4.32E+00	37.7%	5.5%	12.8%
1	F-HCr-L	65	7.5	5%-chrW	6.51E-01	3.79E+00	na	1.10E+01	24.5%	4.2%	14.7%
1	F-oP-L	73	6.56	5%-chrW	8.26E-01	3.70E+00	na	1.10E+01	23.8%	5.3%	18.2%
1	F-FOM-L	89	15.06	**1%-GW**	1.04E+00	1.43E+00	na	7.31E+00	14.6%	10.6%	42.1%
1	SF-raw	92	15.6	**1%-GW**	5.11E+00	1.15E+01	na	2.26E+01	29.3%	13.0%	30.8%
1	LL-pH	106	11.04	1%-chrW	3.08E-03	4.10E-03	na	3.16E-02	10.6%	7.9%	42.9%
1	Cook-Y	108	6.6	5%-chrW	1.11E-04	8.86E-10	na	6.86E-04	0.0%	13.9%	100.0%
1	Glyc-P	111	12.48	**5%-GW**	9.75E+01	1.38E+00	na	5.16E+02	0.2%	15.9%	98.6%
1	Loin-W	120	6.5542	5%-chrW	1.63E-02	1.35E-01	na	2.02E-01	38.2%	4.6%	10.8%
1	LMA-C	120	6.56	5%-chrW	1.64E+00	1.52E+01	na	1.69E+01	45.1%	4.9%	9.8%
1	F-Ham	137	34.4	**1%-GW**	1.15E+00	2.44E+00	na	5.47E+00	27.0%	12.7%	32.0%
1	F-US	180	9.3	5%-chrW	5.59E-01	2.94E+00	na	3.21E+00	43.8%	8.3%	16.0%
1	Ham-W	204	9.08	5%-chrW	2.00E-02	3.71E-02	na	2.24E-01	13.2%	7.1%	35.0%
2	SF-cook	37	8.42	5%-chrW	3.63E+00	2.70E+00	na	1.79E+01	11.1%	14.9%	57.3%
3	SM-pH	1	11.04	1%-chrW	3.13E-03	2.17E-09	na	3.35E-02	0.0%	8.6%	100.0%
3	F-FOM-B	8	6.98	5%-chrW	7.14E-01	1.97E+00	na	4.68E+00	26.7%	9.7%	26.6%
3	F-Ham	13	6.22	5%-chrW	9.48E-01	1.36E+00	na	6.33E+00	15.8%	11.0%	41.0%
3	LMA-US	84	6.02	5%-chrW	1.33E+00	1.50E+01	na	1.34E+01	50.5%	4.5%	8.1%
3	M-FOM-B	89	8.58	5%-chrW	1.36E+00	8.16E+00	na	1.33E+01	35.8%	6.0%	14.3%
3	F-HCr-B	145	12.36	**5%-GW**	8.00E-01	1.08E+00	na	7.38E+00	11.7%	8.6%	42.5%
3	LL-b*	160	9.89	1%-chrW	1.39E-01	2.32E-01	na	1.30E+00	13.9%	8.3%	37.5%
4	F-oP-B	28	5.8	5%-chrW	6.68E-01	3.81E+00	na	7.49E+00	31.9%	5.6%	14.9%
4	pH-45	40	6.812	5%-chrW	2.57E-03	9.49E-04	na	2.73E-02	3.1%	8.3%	73.0%
4	SM-L*	80	6.12	5%-chrW	1.61E+00	1.60E+00	na	1.27E+01	10.0%	10.1%	50.1%
4	SF-cook	89	5.68	5%-chrW	2.37E+00	3.26E+00	na	1.83E+01	13.6%	9.9%	42.1%
4	LMA-US	116	7.12	5%-chrW	1.83E+00	1.53E+01	na	1.30E+01	50.7%	6.1%	10.7%
4	IMF	120	8.5	5%-chrW	4.91E-02	3.37E-02	na	3.70E-01	7.4%	10.8%	59.3%
4	LL-L*	124	6.1	5%-chrW	7.22E-01	1.53E+00	na	7.29E+00	16.0%	7.6%	32.1%
5	LL-L*	100	12.64	**5%-GW**	2.17E+00	2.09E-07	na	7.23E+00	0.0%	23.1%	100.0%
5	Glyc-P	144	9.54	1%-chrW	5.59E+01	7.62E+01	na	4.88E+02	12.3%	9.0%	42.3%
5	M-FOM-L	161	6.72	5%-chrW	1.22E+00	1.11E+01	na	1.37E+01	42.5%	4.7%	10.0%
5	LL-pH	164	5.8	5%-chrW	1.95E-03	5.43E-03	na	3.14E-02	14.0%	5.0%	26.4%
5	IMF	164	12.78	**5%-GW**	3.98E-02	2.25E-02	na	3.79E-01	5.1%	9.0%	63.9%
5	SF-raw	176	5.8	5%-chrW	2.47E+00	1.65E+01	na	2.08E+01	41.5%	6.2%	13.0%
5	F-Ham	209	11.98	**5%-GW**	1.11E+00	1.26E+00	na	6.29E+00	14.6%	12.8%	46.7%
5	F-US	212	16.6	**1%-GW**	6.16E-01	2.87E+00	na	3.18E+00	43.1%	9.3%	17.7%
5	F-FOM-B	212	18.16	**1%-GW**	1.28E+00	1.23E+00	na	4.79E+00	16.8%	17.6%	51.1%
5	F-FOM-L	221	17	**1%-GW**	9.45E-01	1.30E+00	na	7.32E+00	13.5%	9.9%	42.2%
5	F-HCr-B	221	6.56	5%-chrW	8.30E-01	1.59E+00	na	7.09E+00	16.7%	8.7%	34.3%
6	LL-pH	44	8.22	5%-chrW	2.44E-03	6.05E-03	na	3.07E-02	15.4%	6.2%	28.8%
6	LL-a*	54	20.2	**1%-GW**	1.94E-01	6.13E-01	na	7.55E-01	39.2%	12.4%	24.0%
6	LL-L*	60	8.52	5%-chrW	6.80E-01	1.45E+00	na	7.33E+00	15.3%	7.2%	31.9%
6	F-US	64	9.22	1%-chrW	3.83E-01	3.01E+00	na	3.27E+00	45.2%	5.7%	11.3%
6	F-FOM-B	64	7.26	5%-chrW	3.73E-01	2.96E+00	na	4.30E+00	38.7%	4.9%	11.2%
6	LL-b*	64	16.27	**1%-GW**	1.62E-01	3.41E-01	na	1.21E+00	19.9%	9.5%	32.3%
6	F-oP-B	72	11.3	**5%-GW**	6.97E-01	4.38E+00	na	7.15E+00	35.8%	5.7%	13.7%
6	IMF	80	8.67	5%-chrW	2.49E-02	4.98E-02	na	3.71E-01	11.2%	5.6%	33.3%
6	Loin-W	96	7.8052	1%-chrW	1.40E-02	1.31E-01	na	2.05E-01	37.4%	4.0%	9.7%
6	LMA-US	100	11.32	**5%-GW**	1.71E+00	1.48E+01	na	1.34E+01	49.4%	5.7%	10.4%
6	ADG	101	8	1%-chrW	1.04E+03	8.31E+03	1.05E+03	6.89E+03	48.1%	6.0%	11.2%
7	Birth-W	40	9.482	1%-chrW	7.73E-03	3.26E-02	3.92E-02	6.20E-02	23.1%	5.5%	19.1%
7	IMF	48	9.36	1%-chrW	3.07E-02	3.24E-02	na	3.79E-01	7.3%	6.9%	48.7%
7	ADG	49	6.62	5%-chrW	7.80E+02	8.71E+03	1.20E+03	6.70E+03	50.1%	4.5%	8.2%
7	Ham-W	56	6.518	5%-chrW	1.43E-02	5.66E-02	na	2.16E-01	19.8%	5.0%	20.2%
7	F-FOM-B	88	7.9	5%-chrW	4.33E-01	2.98E+00	na	4.25E+00	38.9%	5.7%	12.7%

**7**	**F-oP-B**	88	11.96	**5%-GW**	1.07E+00	3.54E+00	na	7.45E+00	29.3%	8.9%	23.2%
**7**	**M-FOM-L**	109	8.18	5%-chrW	2.57E+00	7.04E+00	na	1.54E+01	28.2%	10.3%	26.7%
**8**	**F-US**	4	13.18	**5%-GW**	4.19E-01	3.07E+00	na	3.18E+00	46.0%	6.3%	12.0%
**8**	**pH-45**	16	5.898	5%-chrW	2.68E-03	6.71E-04	na	2.76E-02	2.2%	8.7%	80.0%
**8**	**Ham-W**	52	13.798	**5%-GW**	1.79E-02	4.22E-02	na	2.20E-01	15.1%	6.4%	29.8%
**8**	**LMA-C**	92	9.56	1%-chrW	2.62E+00	1.34E+01	na	1.73E+01	40.3%	7.9%	16.3%
**9**	**SM-pH**	153	8.22	1%-chrW	3.56E-03	2.32E-09	na	3.30E-02	0.0%	9.7%	100.0%
**9**	**M-FOM-L**	169	7.6	5%-chrW	2.32E+00	7.83E+00	na	1.50E+01	31.2%	9.2%	22.9%
**10**	**Birth-W**	32	12.636	**5%-GW**	1.03E-02	3.06E-02	4.10E-02	6.12E-02	21.4%	7.2%	25.1%
**11**	**Ham-W**	16	10.166	**5%-GW**	1.66E-02	3.90E-02	na	2.24E-01	14.0%	6.0%	29.9%
**11**	**M-FOM-B**	21	22	**1%-GW**	1.35E+00	8.21E+00	na	1.30E+01	36.4%	6.0%	14.1%
**11**	**LMA-US**	36	9.96	**5%-GW**	1.76E+00	1.57E+01	na	1.28E+01	51.9%	5.8%	10.1%
**11**	**M-FOM-L**	37	15.74	**1%-GW**	2.37E+00	8.34E+00	na	1.45E+01	33.1%	9.4%	22.1%
**11**	**F-FOM-L**	141	6.36	5%-chrW	1.18E+00	1.01E+00	na	7.42E+00	10.5%	12.3%	54.0%
**12**	**pH-45**	20	8.82	1%-chrW	2.14E-03	1.15E-03	na	2.74E-02	3.8%	7.0%	65.0%
**12**	**F-HCr-B**	25	5.3	5%-chrW	8.61E-01	1.91E+00	na	6.86E+00	19.8%	8.9%	31.1%
**13**	**pH-45**	36	6.624	5%-chrW	3.67E-03	1.76E-04	na	2.71E-02	0.6%	11.9%	95.4%
**13**	**M-FOM-B**	77	10.98	1%-chrW	1.13E+00	8.25E+00	na	1.33E+01	36.4%	5.0%	12.0%
**13**	**M-FOM-L**	77	10.3	1%-chrW	1.43E+00	9.94E+00	na	1.42E+01	38.8%	5.6%	12.6%
**13**	**Loin-W**	80	19.114	**1%-GW**	2.70E-02	1.25E-01	na	1.99E-01	35.6%	7.7%	17.7%
**13**	**ADG**	85	5.92	5%-chrW	6.60E+02	8.46E+03	1.08E+03	6.92E+03	49.4%	3.9%	7.2%
**13**	**LMA-US**	88	10.44	1%-chrW	2.29E+00	1.54E+01	na	1.27E+01	50.7%	7.5%	12.9%
**13**	**F-oP-L**	189	6.56	5%-chrW	1.96E+00	2.04E+00	na	1.14E+01	13.3%	12.7%	49.0%
**14**	**M-FOM-L**	33	5.92	5%-chrW	1.61E+00	7.69E+00	na	1.55E+01	30.9%	6.5%	17.3%
**14**	**SF-cook**	33	6.42	5%-chrW	1.65E+00	6.34E+00	na	1.68E+01	25.5%	6.6%	20.7%
**14**	**LMA-C**	56	9.44	1%-chrW	2.31E+00	1.48E+01	na	1.67E+01	43.8%	6.8%	13.5%
**15**	**ADG**	17	8.9	1%-chrW	1.40E+03	8.13E+03	9.82E+02	6.83E+03	46.9%	8.1%	14.7%
**15**	**F-US**	32	26.8	**1%-GW**	5.60E-01	3.52E+00	na	2.81E+00	51.1%	8.1%	13.7%
**15**	**SF-raw**	45	8.5	5%-chrW	4.77E+00	1.46E+01	na	2.08E+01	36.4%	11.9%	24.6%
**15**	**M-FOM-L**	141	5.74	5%-chrW	3.14E+00	8.46E+00	na	1.43E+01	32.7%	12.1%	27.1%
**16**	**F-oP-L**	1	5.54	5%-chrW	7.19E-01	3.65E+00	na	1.11E+01	23.5%	4.6%	16.5%
**16**	**F-HCr-B**	1	4.8	5%-chrW	4.19E-01	2.46E+00	na	6.79E+00	25.5%	4.3%	14.5%
**16**	**F-oP-B**	8	5.56	5%-chrW	4.77E-01	4.18E+00	na	7.39E+00	34.7%	4.0%	10.2%
**16**	**SM-a***	8	7.63	5%-chrW	3.67E-01	2.07E-01	na	2.76E+00	6.2%	11.0%	63.9%
**16**	**F-Ham**	9	8.46	5%-chrW	5.70E-01	2.18E+00	na	6.07E+00	24.7%	6.5%	20.7%
**16**	**SM-b***	12	6.168	5%-chrW	2.48E-01	3.07E-01	na	2.26E+00	10.9%	8.8%	44.6%
**16**	**SF-raw**	16	10.98	**5%-GW**	3.76E+00	1.74E+01	na	1.96E+01	42.6%	9.2%	17.8%
**16**	**Cook-Y**	20	8.16	1%-chrW	6.77E-05	9.00E-05	na	6.44E-04	11.2%	8.4%	42.9%
**16**	**F-US**	36	6.6	5%-chrW	2.93E-01	3.40E+00	na	3.09E+00	50.1%	4.3%	7.9%
**16**	**SF-cook**	49	5.22	5%-chrW	1.31E+00	5.84E+00	na	1.73E+01	23.9%	5.4%	18.3%
**16**	**F-FOM-B**	64	12.74	**5%-GW**	7.00E-01	2.77E+00	na	4.21E+00	36.1%	9.1%	20.2%
**16**	**F-FOM-L**	81	7.86	5%-chrW	8.40E-01	1.67E+00	na	7.22E+00	17.1%	8.6%	33.5%
**17**	**SM-a***	48	8.494	5%-chrW	2.44E-01	6.05E-01	na	2.56E+00	17.8%	7.2%	28.7%
**18**	**IMF**	20	7.5	5%-chrW	5.97E-02	8.46E-07	na	3.84E-01	0.0%	13.5%	100.0%
**18**	**F-oP-B**	28	7.4	5%-chrW	9.89E-01	3.21E+00	na	7.67E+00	27.0%	8.3%	23.6%
**18**	**LMA-C**	40	4.9	5%-chrW	2.05E+00	1.45E+01	na	1.71E+01	43.1%	6.1%	12.4%
**18**	**F-oP-L**	73	5.72	5%-chrW	1.11E+00	2.89E+00	na	1.13E+01	18.9%	7.2%	27.7%
**18**	**LL-pH**	80	4.68	5%-chrW	1.46E-03	6.62E-03	na	3.09E-02	17.0%	3.7%	18.0%
**18**	**LL-L***	80	13.24	**5%-GW**	7.44E-01	1.02E+00	na	7.50E+00	11.0%	8.0%	42.2%
**18**	**F-HCr-B**	81	5.38	5%-chrW	8.31E-01	1.95E+00	na	6.89E+00	20.2%	8.6%	29.9%
