# Supplementary material for: Joint analysis of quantitative trait loci and major-effect causative mutations affecting meat quality and carcass composition traits in pigs
Source: BMC Genet. 2011 Aug 29;12:76. doi: 10.1186/1471-2156-12-76 (PMC3175459; doi:10.1186/1471-2156-12-76)
Supplement: Additional file 6 — Chromosome-wise significance thresholds. Chromosome-wise thresholds as quantiles of the distribution of QTL detection statistical test used when applied to phenotypes simulated under the null hypothesis (polygenic additive model, H0: h2 = 30%). [file 1471-2156-12-76-S6.PDF]

Chromosome-wise LRT significance thresholds

	Selected quantiles of LRT distribution under null hypothesis 5,000 simulated datased (305 animals, 4 half sibs families, h^2 0.3)			4 test chromosomes on full size datasets 1,000 simulated datasets (760 animals; h^2 0.3)
**SSC**	**5%**	**1%**	**0.285%**	**5%**
**1**	6.40	9.49	11.88	6.41
**2**	5.73	8.86	10.94	-
**3**	5.73	9.19	11.87	-
**4**	5.40	8.63	10.89	-
**5**	5.40	8.23	10.94	-
**6**	5.68	8.68	11.06	5.81
**7**	6.04	9.29	11.84	-
**8**	5.84	8.37	10.83	-
**9**	5.43	8.26	10.89	-
**10**	5.15	7.84	10.46	-
**11**	5.12	7.72	9.92	-
**12**	5.00	7.94	9.37	-
**13**	5.67	8.39	11.72	-
**14**	5.57	8.24	10.73	-
**15**	5.49	8.94	11.39	5.49
**16**	4.80	8.04	10.42	-
**17**	5.33	8.92	11.92	-
**18**	4.63	7.70	10.68	4.73
